# Chemically Driven Division of Protocells by Membrane Budding

**DOI:** 10.1021/jacs.4c08226

**Published:** 2024-11-27

**Authors:** Pablo Zambrano, Xiaoyao Chen, Christine M. E. Kriebisch, Brigitte A. K. Kriebisch, Oleksii Zozulia, Job Boekhoven

**Affiliations:** † Department of Bioscience, Technical University of Munich, Lichtenbergstrasse 4, 85748 Garching, Germany

## Abstract

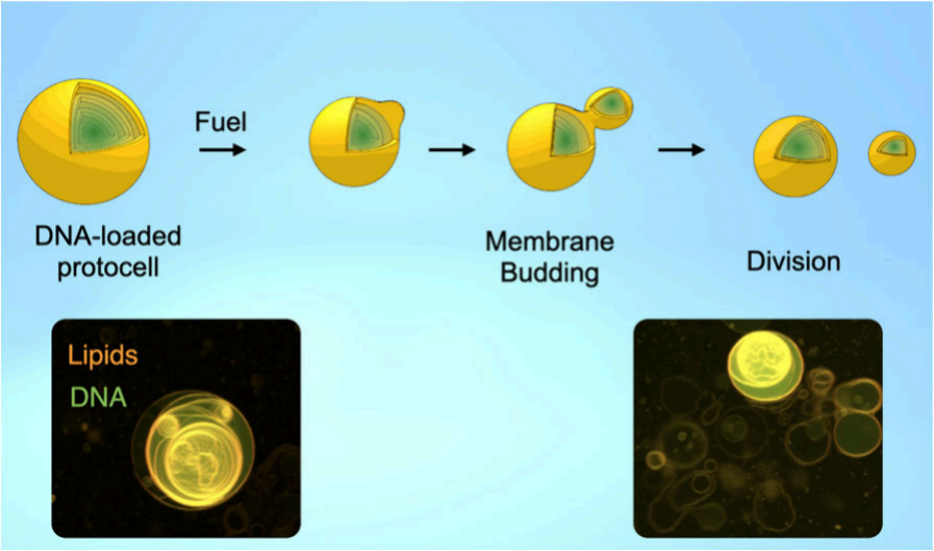

Division is crucial for replicating biological compartments and, by extension, a fundamental aspect of life. Current studies highlight the importance of simple vesicular structures in prebiotic conditions, yet the mechanisms behind their self-division remain poorly understood. Recent research suggests that environmental factors can induce phase transitions in fatty acid-based protocells, leading to vesicle fission. However, using chemical energy to induce vesicle division, similar to the extant of life, has been less explored. This study investigates a mechanism of vesicle division by membrane budding driven by chemical energy without complex molecular machinery. We demonstrate that, in response to chemical fuel, simple fatty acid-based vesicles can bud off smaller daughter vesicles. The division mechanism is finely controlled by adjusting fuel concentration, offering valuable insights into primitive cellular dynamics. We showcase the robustness of self-division across different fatty acids, retaining encapsulated materials during division and suggesting protocell-like behavior. These results underscore the potential for chemical energy to drive autonomous replication in protocell models, highlighting a plausible pathway for the emergence of life. Furthermore, this study contributes to the development of synthetic cells, enhancing our understanding of the minimal requirements for cellular life and providing a foundation for future research in synthetic biology and the origins of life.

## Introduction

Life depends fundamentally on its ability to replicate. Whether symmetrically or asymmetrically, the minimal unit of life—the cell—inevitably must divide. Even at the origin of life, the division of the protocells was critically important. Such protocells are thought to date back to simple structures, such as droplets or membranous vesicles composed of amphiphilic molecules, likely precursors of modern complex cellular architectures.^1−3^ Despite extensive studies on models of these structures, the mechanisms by which such simple vesicular structures could generate offspring through self-division, especially in the absence of the complex molecular machinery typical of contemporary life forms, remain poorly understood.^4^ In contrast, modern cell division within phospholipid compartments is highly regulated and involves intricate networks of macromolecular complexes, enzymes, and proteins.^5^ This process starkly contrasts early protocellular systems, which, lacking such complex components, must have relied on fundamentally different mechanisms for replication and division.^6−8^

The division of compartments using chemical energy without the mediation of complex molecular machinery is rarely described, underscoring an important gap in our understanding of primitive life processes.^9^ Recent advances have highlighted how environmental factors such as temperature, pH, and UV light induce phase transitions in fatty acid-based protocells, leading to vesicle fission and daughter vesicles.^4,7,10,11^ These findings suggest that environmental conditions may have played a critical role in replicating early protocells. Furthermore, studies have shown that self-assembling single-chain amphiphiles, likely available in the prebiotic environment, played a fundamental role in the advent of primitive cell cycles.^12,13^ Moreover, research on self-reproducing catalytic micelles supports the idea that amphiphiles could facilitate early cellular functions.^14,15^ Previous studies have demonstrated the assembly and formation of lipid bilayers from nonlipid precursors, providing a model for membrane biogenesis in synthetic systems.^16−18^ In our work, we expand on this concept by using carbodiimide-fueled reactions to induce vesicle transformation, including membrane budding leading to division.

**Scheme 1:**
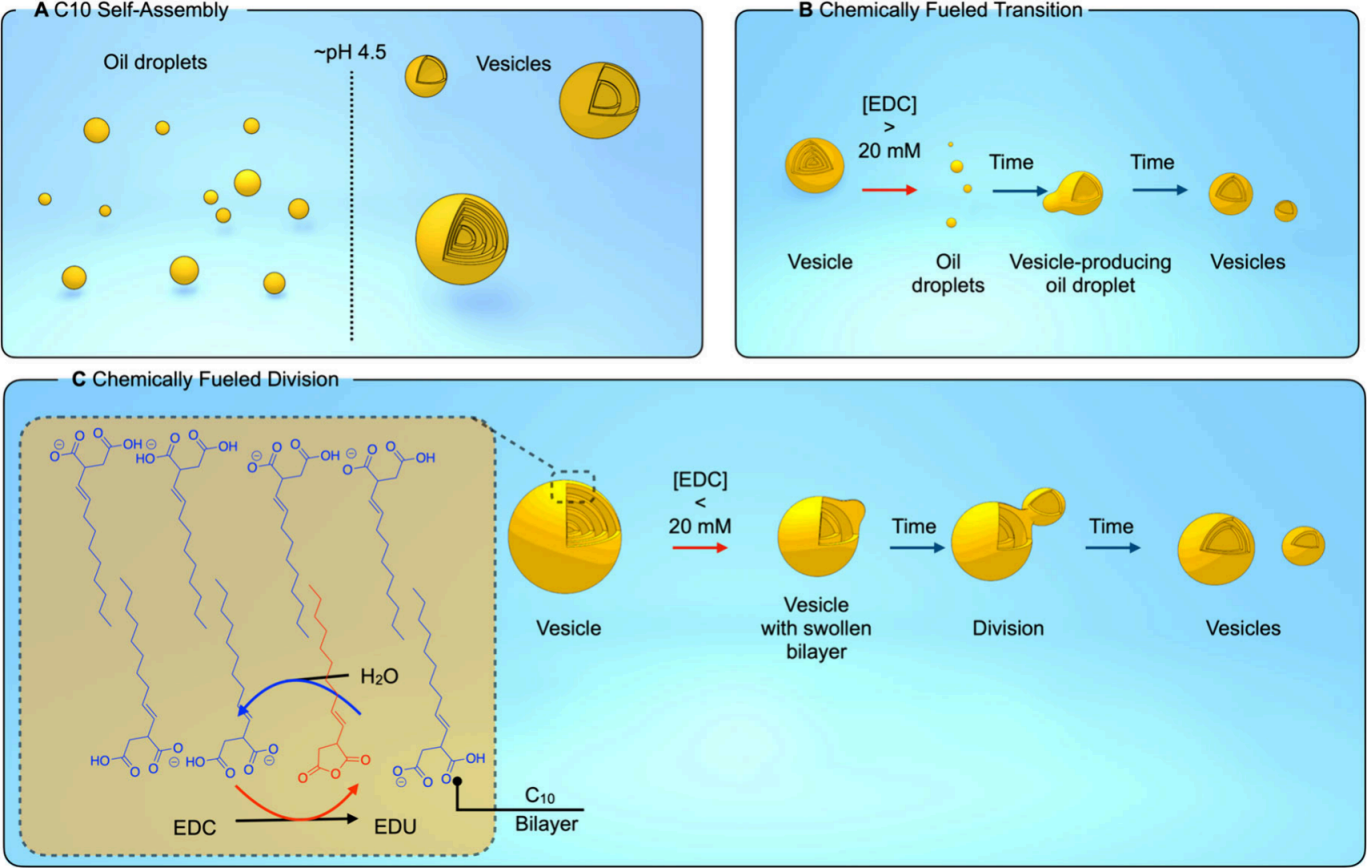
Vesicle Formation and Self-Division Mechanism. (A) C10 Forms Oil Droplets below pH 4.5 and Vesicles above pH 4.5. (B) High EDC Concentration Converts Vesicles to Oil Droplets, Producing New Vesicles. (C, Right) Low EDC Concentrations Only Partially Convert the Bilayer, Resulting in a Swollen Bilayer. With Time, the Oil Hydrolyzes and Buds of New Vesicles. (C, Left) The Chemical Reaction Cycle Responsible for the Behavior.

This work introduces a mechanism for membranous vesicle self-division facilitated by converting a sacrificial carbodiimide with high chemical potential. We use a chemical cycle driven by this carbodiimide as a condensing agent to induce structural changes in vesicles composed of a succinic acid derivative.^19−24,35,36^ The succinic acid derivative forms vesicles at a pH above 4.5, while it exists as oil droplets at lower pH (Scheme 1a). The reaction cycle involves the partial conversion of the amphiphilic diacid-molecule into transient oily anhydride molecules (Scheme 1b,c), followed by the rapid hydrolysis back to the original succinic acid amphiphile. Interestingly, this dynamic process leads to the local production of excess amphiphilic diacid molecules that assemble to form newly budding vesicles. By carefully controlling the conversion, we can direct the formation of these vesicles—either they are completely converted to oil droplets and produce new vesicles, or they are partially converted, leaving the vesicles intact and subsequently budding of vesicles. We explore the transfer of contents from progenitor vesicles to the newly formed compartments. By elucidating this mechanism of chemically driven self-division, our work aims to deepen our understanding of primitive cellular functions and contribute to the emerging field of synthetic biology, where creating cells with autonomous division capability remains a major challenge.^25−27^

Finally, we extended our study of the self-dividing mechanism of our initial amphiphilic molecule to include prebiotic vesicles composed of decanoic acid, a molecule believed to have been abundant on the early Earth.^12,28−30^ This extension of our investigation demonstrates the robustness of the mechanism of self-division in different amphiphilic systems, suggesting that such processes could have played a crucial role in the self-replicative behaviors of early protocells. This aspect of our study not only tests the versatility of our proposed mechanism in diverse lipid-like environments but also enhances our understanding of the types of molecular assemblies that could have facilitated the emergence of life.^27,31^ Our work also lays the conceptual foundation for developing synthetic cells capable of autonomous self-replication, reflecting the most basic properties of life.^32−34^

## Results and Discussion

### A Fueled Morphological Transition toward Smaller Vesicles

For our amphiphiles, we use a succinic acid–based fatty acid. In previous work, we found that this molecule is readily converted to anhydride using chemical fuel.^20^ Specifically, we used 2-decen-1-yl-succinic acid (C10, Scheme 1C) at 65 mM in 200 mM MES buffer, the conditions throughout the work. We found that the behavior of C10 varies drastically with pH, which was investigated by confocal microscopy and turbidity measurements (Figure S1).^34^ At pH below 4.0, we observed that C10 predominantly forms oil droplets, whereas at pH above 4.5, unilamellar and multilamellar vesicles emerge clearly in solution. We measured an apparent p*K*_a_ for C10 to be 5.41 (Figure S2) and can, therefore, conclude that C10 transitions from vesicles to oil droplets when its carboxylates become protonated. This behavior aligns with other fatty acids that tend to form oil droplets at pH values below their apparent p*K*_a_ and transition to form vesicles near their apparent p*K*_a_.^12,33,34^ Following our confocal microscopy findings, we focused on working at a pH of 4.9 to ensure a constant presence of vesicles. Additionally, we found that the critical vesicle concentration is above 1 mM (Figure S3). Additional confocal experiments were conducted to determine the optimal concentration of C10 at which vesicles were formed that were predominantly greater than 1 μm in size form (Figure S4–S6). FRAP analysis confirmed the membrane fluidity of the vesicles with a calculated diffusion coefficient and recovery time (Figure S7). Furthermore, we observed selective labeling of C10 vesicles by Merocyanine-540—a dye that selectively binds vesicles over oil droplets—with no interaction with anhydride oil droplets (Figure S8).

**Figure 1:**
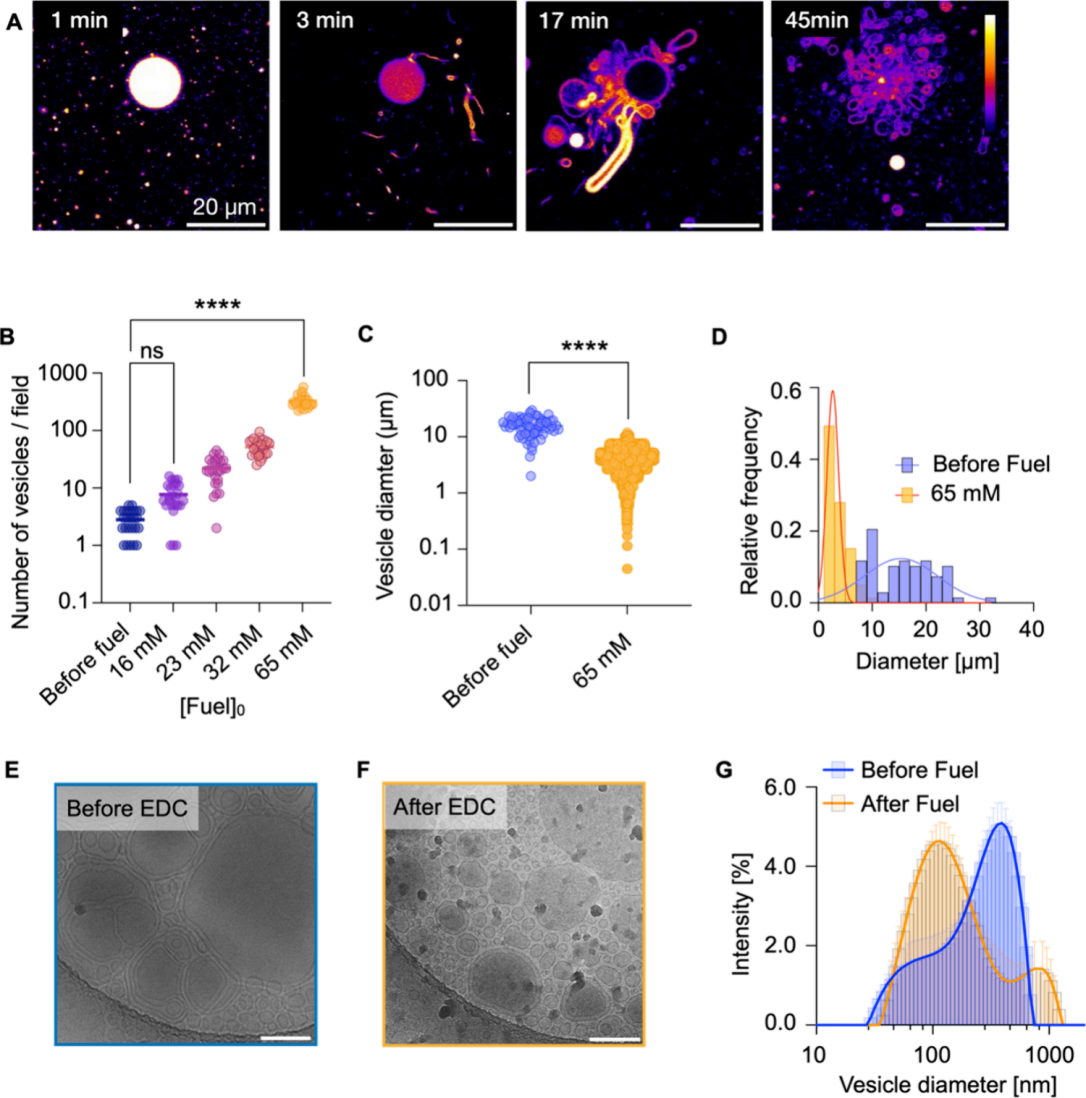
(A) Confocal micrographs of the morphological transition of a C10 multilamellar vesicle (65 mM) in response to EDC 23 mM at pH 4.9. The vesicles were stained with 2 μM Nile Red to highlight structural features. (B) The number of vesicles generated after adding different EDC concentrations. *n* = 25 (per condition, images analyzed). ANOVA was used to identify significant group differences, *p* < 0.0001****. Variance homogeneity was confirmed by Brown–Forsythe and Bartlett’s tests, both *p* < 0.0001****. The model explains 91.92% of the variance (*R*^2^ = 0.9192). (C) Average diameter of vesicles generated after addition of EDC 65 mM to a C10 solution (65 mM). Unpaired *t* test analysis between ‘Before fuel’ and ‘EDC 65 mM’, *p* < 0.0001****. *n* = 68 for ‘Before fuel’ and *n* = 8131 for ‘EDC 65 mM’. (D) Comparative histogram and Gaussian fits of vesicle size distribution after addition of EDC 65 mM to a C10 vesicle solution (65 mM). Bars represent vesicle size histograms for the ‘Before fuel’ group (*n* = 68, blue) and ‘65 mM EDC’ group (*n* = 8131, orange). Lines represent Gaussian fits: ‘Before fuel’ group (blue): amplitude 0.1236, mean 15.36 μm, SD 6.820 μm; ‘65 mM EDC’ group (orange): amplitude 0.5916, mean 2.684 μm, SD 1.103 μm. (E) Cryo-TEM micrographs of C10 vesicles (65 mM; MES buffer 200 mM; pH 4.9) before and after (F) addition of EDC 23 mM. Scale bar: 200 nm. (G) Results of dynamic light scattering (DLS) analysis of 400 nm multilamellar C10 vesicles obtained by extrusion (blue; PDI = 0.34) after exposure to 23 mM EDC (orange; PDI = 0.38). Error bars represent standard deviations (SD) with *n* = 3.

Our reaction cycle uses a condensing agent (1-ethyl-3-(3-(dimethylamino)propyl)carbodiimide hydrochloride (EDC or fuel) as a chemical fuel to convert C10 to its corresponding anhydride (activation). The anhydride is unstable in the aqueous medium and hydrolyzes to the precursor (deactivation). Therefore, when a finite amount of fuel is added, the anhydride emerges and disappears again as the fuel is depleted.^19,21,22^ Using confocal microscopy, we observed remarkable transformations upon adding EDC to a vesicle solution of 65 mM C10— in the first 60 s after adding 23 mM EDC, we observed a marked increase in the fluorescence intensity of the vesicles. This increase is attributed to the solvatochromic dye Nile Red, whose quantum yield is greater in oily phases than in lipid bilayers. Moreover, minutes after the addition of EDC, the lumen of the vesicles was no longer observed (Figure 1A). We conclude that the vesicles were almost all completely converted to oil droplets as a result of the partial conversion of the C10 into its corresponding anhydride. Moreover, a wide range of droplet sizes was observed, pointing to their rapid fusion. In the following minutes, the fluorescence intensity decreased again, and new membranous structures formed on the surface of the oil droplets. The process was particularly clear from large oil droplets pinned to the microscope slide’s glass surface. These new vesicles were initially elongated and attached to the droplet from which they originated, then began to grow and, after 15 min, adopted unilamellar and multilamellar forms. However, these new vesicles remained attached to the mother vesicle until, finally, the original multilamellar vesicles reduced in size and disappeared completely, leaving a set of separate, individual vesicles (Figure 1A, Figure S9, Movie S1–S2). In addition, we monitored the pH of the reaction cycle throughout the experiment and ensured pH fluctuations did not influence our observations (Supplementary Table 4).

We hypothesize that the condensing agent converts a large amount of the amphiphiles of the vesicles into their corresponding anhydrides. We assume these anhydrides, along with the remaining amphiphiles, form oil droplets that rapidly fuse into a mixture of droplets that are polydisperse in size. As the fuel is rapidly depleted, deactivation through hydrolysis dominates and recovers the amphiphiles locally at the surface of the oil droplets. The high concentration of amphiphiles produces new vesicles from the oil droplets that pinch off into individual compartments.

We quantified the production of new vesicles using confocal microscopy by imaging the C10 vesicles before and after adding varying concentrations of EDC, with at least 25 images captured per condition. We observed that the number of vesicles increases significantly with higher EDC concentrations (Figure 1B). For example, with 16 mM EDC, the number of vesicles doubles compared to the initial amount observed before fuel addition. As expected, we also observed a significant reduction in the size of vesicles formed in response to EDC compared to the parent vesicles (Figure 1C–D). Specifically, while the average diameter of the stock vesicles was approximately 15.8 + 5.6 μm, the vesicles formed after EDC addition showed an average diameter of only 3.6 ± 2.0 μm (Figure S10). These changes reflect a dynamic process of vesicle reorganization and division driven by the addition of EDC.

To investigate whether the conversion of larger vesicles into smaller ones via a morphological transition involving oil droplets depends on the initial vesicle size, we conducted experiments with C10 vesicles prepared by extrusion through a 400 nm filter. Before fuel addition, cryo-TEM micrographs revealed vesicles that were polydisperse in size, ranging from a diameter of 50 nm to hundreds of nm, despite the extrusion at 400 nm (Figure 1E). Furthermore, DLS confirmed the polydisperse nature with a broad peak centered around 400 nm (Figure 1G). Nevertheless, 1.5 h after adding 23 mM EDC, cryo-TEM micrographs show a marked decrease in vesicle size (Figure 1F). DLS analysis further corroborated these observations—following the EDC exposure, the DLS data reveals a significant shift in the size distribution toward smaller vesicle sizes, around 100 nm (Figure S11). Additionally, a secondary peak around 1000 nm suggests the presence of larger vesicular structures pointing toward the fusion of the droplets or vesicles, which could be observed by brightfield microscopy (Figure S14–S15). Finally, we investigated the behavior with 100 nm vesicles using confocal microscopy and Cryo-EM, supported by dynamic light scattering (DLS) analysis, which also pointed toward the formation of smaller vesicles (Figure S12). These findings generally align with recent studies suggesting membrane phase transitions driven by environmental fluctuations can generate daughter protocells with reorganized contents.^4,10^ In contrast to those studies, where phase transitions are induced by temperature and pH fluctuations, we show a fuel-driven chemical conversion can induce a morphological transition that leads to smaller vesicles.

### Controlled Activation Leading to Vesicle Self-Division by Budding

**Figure 2:**
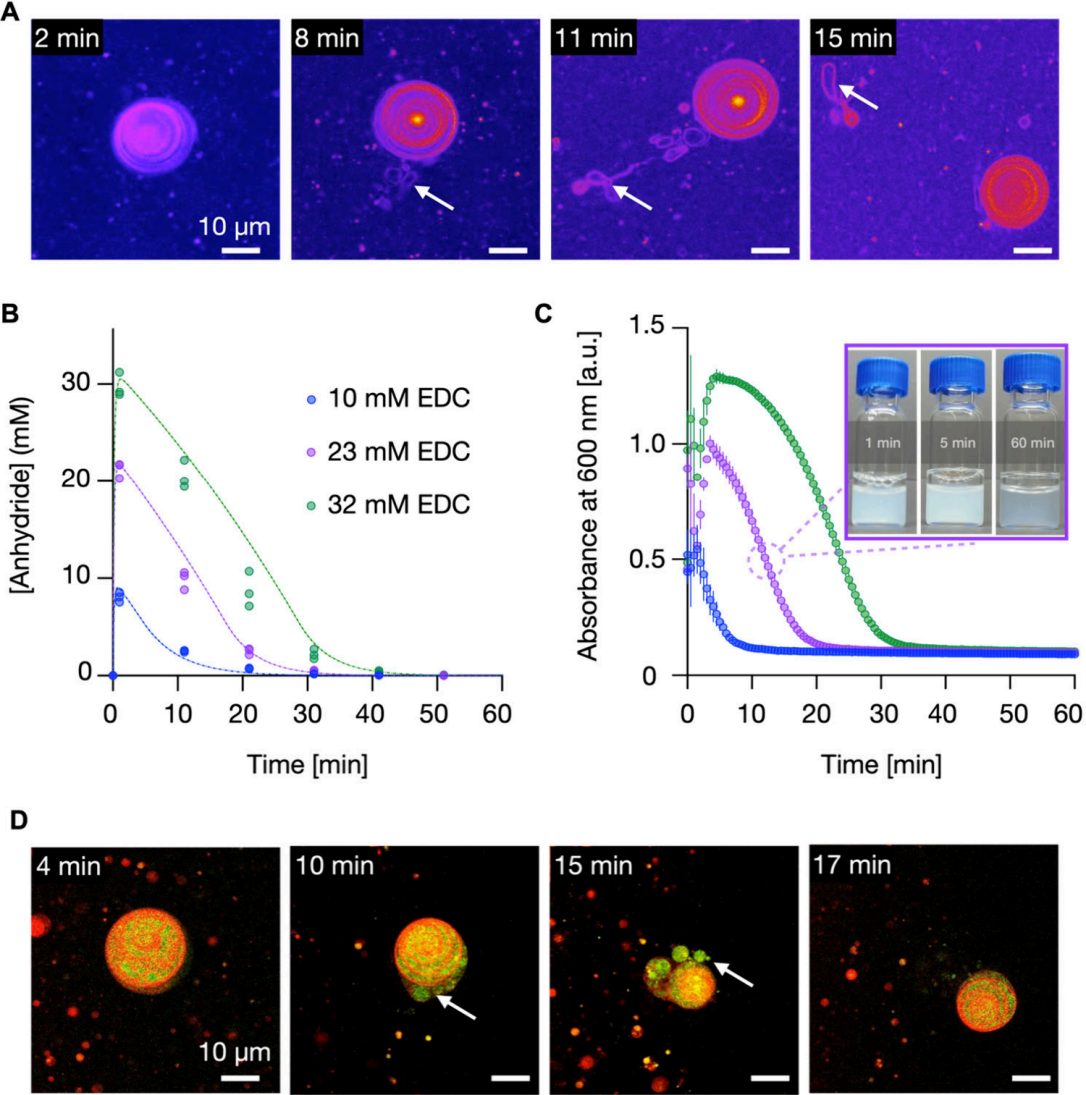
(A) Generation of “daughter” vesicles from the surface of a multilamellar vesicle. Conditions: C10 65 mM precursor (Nile Red dye; 2 μM), EDC 10 mM, MES buffer 200 mM, pH 4.94. The arrow indicates the location of the emergence of the “daughter” vesicle and its trajectory until it detaches from the “mother” vesicle. (B) C10 anhydride concentration as a function of time (symbols; triplicates) and kinetic model (line) for a 65 mM precursor in the presence of different EDC concentrations. (C) Turbidimetry measurements at 600 nm for a 65 mM precursor at different EDC concentrations. Error bars represent standard deviations (SD) with *n* = 3. Inset: visual observation of turbidity changes over time. (D) Division process of multilamellar vesicles (labeled with 20 μM Nile Red) with encapsulated material (labeled-DNA; 1.5 μM) in the presence of 10 mM EDC. The sequence reveals the formation of smaller daughter vesicles, maintaining the integrity of the encapsulated material.

The data above show chemical fuel can induce a morphological transition from vesicles to oil droplets to smaller vesicles. However, the intermediate oil droplets are destroying the original vesicles, which is clearly not division. We hypothesized that smaller amounts of fuel could prevent the complete collapse of the vesicles into oil droplets and, instead, lead to a bilayer swollen with some oil. The local production of fatty acids through the oil’s hydrolysis could bud off new vesicles. Such a process would be closer to self-division through budding. Following this approach, we added less fuel to the multilamellar vesicles (10 mM EDC, 65 mM C10, pH 4.9). Data obtained by confocal microscopy showed that activation of the precursor results in a moderate increase in fluorescence intensity of the vesicle surface, indicating a minor conversion of the precursor to anhydride (Figure 2A; Movie S3). Importantly, the multilamellar vesicles mostly stayed intact instead of fully converting to an oil droplet. Excitingly, after 8 min, we observed the formation of thin, unilamellar membranous structures on the surface of the multilamellar vesicles, which slowly grew into elongated compartments (Movie S3). These new membranous structures remained attached to the mother vesicle for more than 7 min until they finally separated from the mother vesicle. This process of producing daughter vesicles continued for 15 min. The division behavior was heterogeneous as some vesicles produced many small daughter vesicles, whereas others produced only a few. The heterogeneity is likely a result of localized variations in the concentration of reactants and the structural integrity of the vesicle membrane. Observing these varying growth rates provides valuable insights into the mechanistic aspects of vesicle division, highlighting the complex interplay between chemical kinetics and membrane dynamics (Figure S16, Movies S4, S5).

To understand the chemical kinetics behind the self-division, we used high-performance liquid chromatography (HPLC) to examine the evolution of the concentrations using 65 mM C10 and 10 mM EDC. We monitored the concentration of C10, its corresponding anhydride, and EDC (Figure 2B; Figure S17) and found an initial rapid increase in the anhydride concentration simultaneously with a consumption of the EDC. After this initial increase, the anhydride concentration steadily decreased until it disappeared within 30 min. This linear decay can be explained by a self-protection mechanism that we previously documented.^20,22^ In this mechanism, the anhydride is encapsulated and isolated from water, leading to the hydrolysis to occur exclusively on the anhydride that remains in the aqueous medium, resulting in a linear decrease of the anhydride until all droplets have completely dissolved. Such a pattern establishes that the decomposition rate is directly linked to the solubility of the anhydride, which is constant. Using a kinetic model, we could accurately fit and predict the kinetics described in our reaction cycle (Supporting Information, Methods).

In parallel, we monitored the evolution of the optical density at 600 nm by absorbance measurements in response to various amounts of fuel (Figure 2C). The data revealed a rapid, slight increase in turbidity during the first few minutes after the addition of 10 mM EDC, followed by a gradual decrease in turbidity. We assume the increase in the turbidity is a result of the slight, transient swelling of the membrane by the anhydride. Minutes later, the turbidity decreased back to the original level. The process correlates nicely with the behavior of the system. With 10 mM of EDC, the division was mostly visible by microscopy in the first 10 min, backed by the anhydride and turbidity decaying in the first 10 min. When more EDC was added, the turbidity increased strongly, indicating the formation of oil droplets in line with our earlier observations (Figure 1). Finally, we carried out similar experiments with 100 nm vesicles and monitored the vesicle size by cryo-TEM and DLS which further corroborated division (Figure S12). Thus, we conclude that division through budding can be induced if only small amounts of EDC are added.

The combined data points at the partial conversion of the succinic-acid–based amphiphiles into their corresponding anhydride. As we used small amounts of fuel, the vesicles remained intact, and we hypothesize the anhydride oil swells the bilayer. As the anhydride hydrolyzes either in the bilayer or in its vicinity, it is converted into the original amphiphile. The excess amphiphiles restructure the vesicles either by budding off new vesicles, increasing the original bilayer’s size, or increasing the number of lamella of the vesicle. The absence of complex biomolecular machinery is attractive in this work, but it comes with the downside that the division process is heterogeneous.

To refer to the production of new vesicles as a division, we ensured that the contents of the mother vesicles were transferred to the daughter vesicles instead of the production of new vesicles outside of the original one. Previous studies have shown that maintaining the integrity of encapsulated materials during vesicle division is a significant challenge, as many systems lose their internal contents due to membrane instability.^6−8,13^ Thus, we studied various techniques to encapsulate molecules within our multilamellar vesicles (See Supporting Information–Methods) and found that they can retain fluorescently labeled DNA (Figure S19). These vesicles divided in response to 10 mM EDC by budding off daughter vesicles smaller than the parent vesicle, which contained some of the encapsulated labeled DNA after the division. Confocal microscopy experiments (Figure 2D; Movie S6) captured this process, where vesicles with Nile Red-stained membranes and encapsulated labeled DNA were observed at various time points after adding 10 mM EDC. By 10 min, smaller daughter vesicles formed on the mother vesicle’s surface, clearly retaining the encapsulated DNA within their interiors. By 15 min, these daughter vesicles slowly detached from the mother vesicle, as shown in the images. Finally, at 17 min, the mother vesicle was observed without the daughter vesicles, which had dispersed into the solution and moved out of the focal plane. This capacity to produce smaller daughter vesicles while preserving the encapsulated contents could have profound implications for developing synthetic cells and studying protocell evolution. We demonstrated that the process could be repeated multiple times. As shown in Figure S18, the iterative injection of EDC into C10 multilamellar vesicles resulted in a dynamic response, observed through the periodic increase and recovery of absorbance. These behaviors provide a framework for designing protocellular systems capable of controlled division, which is key to replicating content and functionality in synthetic cells.

Finally, to ensure the division through budding is not a result of the solutions’ inhomogeneous mixing, we added a control experiment in which the fuel was added heterogeneously. We found the collapse of the vesicles into oil droplets—a behavior very different from the controlled addition and mixing of the fuel (Figure S20).

**Figure 3:**
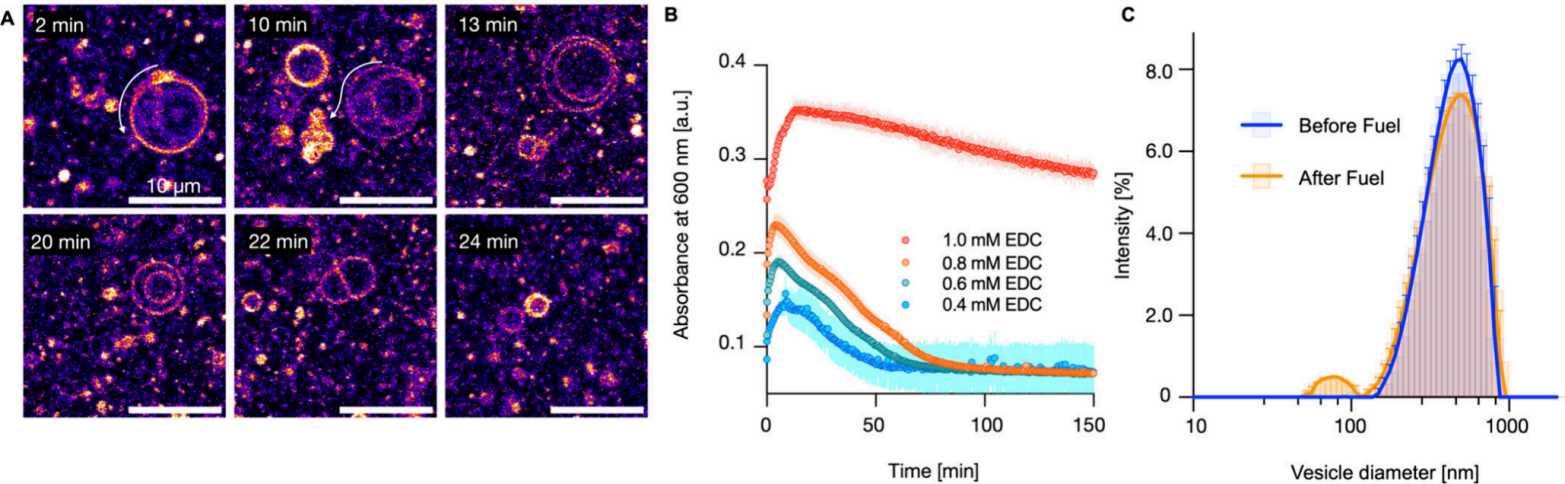
(A) Confocal micrographs showing the division sequence of decanoic acid (DA) vesicles (50 mM; pH 6.8) upon exposure to 0.4 mM EDC. Over time, the vesicle structure becomes disrupted, leading to the formation of smaller daughter vesicles. Dye: Nile Red 2 μM.. (B) Turbidimetry measurements at 600 nm for DA vesicles (50 mM) in MES buffer (200 mM, pH 6.8) with varying EDC concentrations (0.4–1.0 mM). (C) Dynamic light scattering (DLS) analysis of DA vesicles (400 nm multilamellar vesicles obtained by extrusion). The blue curve represents the size distribution of initial vesicles (PDI = 0.28). In contrast, the orange curve shows the size distribution after exposure to 0.4 mM EDC (40 min; PDI = 0.31). Error bars represent standard deviations (SD) with *n* = 3.

We extended our study to decanoic acid (DA), which is considered more prebiotically plausible than C10 and has been much more studied.^26,28,37−40^ First, we determined the critical concentration for vesicle formation (CVC ∼ 40 mM) and the optimal pH for vesicle formation (Figure S21–S24).^4,12,27,41^ At a concentration of 50 mM DA in 0.2 M MES buffer at pH 6.8, the vesicles were numerous and generally not exceeding 50 μm. Unlike the large multilamellar vesicles of C10, DA vesicles were predominantly unilamellar and more abundant (Figure S24). We attributed the slight differences in the behavior of the lipids to the difference in their headgroup—a succinic acid versus simple aliphatic acid. We then investigated the behavior of these vesicles upon the addition of EDC (Figure 3A). In line with the data on C10, at 2 min after 0.4 mM EDC addition, the Nile Red in the vesicle membrane exhibited increased brightness pointing to the intermolecular anhydride formation. Numerous smaller vesicles in the vicinity also transformed into anhydride droplets (bright points). Additionally, EDC induced an accumulation of anhydride in a specific region of the membrane (indicated by the arrow), which, after 10 min, hydrolyzed and formed smaller daughter vesicles. These daughter vesicles detached from the mother vesicle and dispersed into the solution (arrows). At 13 min, the mother vesicle underwent internal membrane reorganization, eventually leading to complete division by 24 min (Movie S7), demonstrating that the division mechanism explored with C10 also functioned effectively with decanoic acid. This finding is significant as it shows the robustness of the condensing agent-induced division mechanism across different amphiphilic systems. After adding 0.4 mM EDC, the number of DA vesicles per field increased significantly, rising from an average of 19 to over 30 vesicles (Figure S25). This increase plateaued with higher concentrations, as observed at 0.6 and 0.8 mM EDC, indicating a saturation point beyond which the vesicle count did not change. In parallel, the vesicle diameter decreased markedly after adding 0.4 mM EDC, with the median size dropping from 7.9 to 4.6 μm. This reduction in vesicle size also resulted in a narrower distribution, highlighting that EDC not only promotes vesicle formation but also drives the formation of smaller vesicles in a controlled manner.

In contrast to C10, much less EDC was required to induce a response by the system. For example, turbidimetry measurements at 600 nm with varying EDC concentrations show that as little as 0.4 mM EDC is sufficient to increase the turbidity (Figure 3B) compared to the 10 mM EDC required to induce a response for C10 vesicles (Figure 2B, Figure S26). We explain the drastic difference between the two seemingly similar amphiphiles by the drastically different nature of the anhydride. Specifically, we expect the anhydride of C10 to be more water-soluble than its DA anhydride counterpart—the anhydride of C10 forms an intramolecular anhydride. Thus, the number of carbons does not increase. In contrast, DA can only form an intermolecular anhydride; thus, its carbon number doubles upon converting into its anhydride, drastically decreasing solubility. As a result, less anhydride is needed to induce a response. In line with the C10 amphiphiles, the turbidity initially rose and leveled off over an hour. Notably, the pH of the solution remained constant throughout the reaction, ensuring that the observed changes were due to the chemical processes induced by EDC rather than pH fluctuations (Table S5). Finally, we explored the reaction in nanometric DA vesicles obtained by extrusion (400 nm). Dynamic light scattering (DLS) analysis quantified the changes in size of these 400 nm multilamellar DA vesicles in the presence of EDC (Figure 3C). After extrusion, the vesicles showed a relatively broad peak around 400 nm. One hour after the addition of EDC, the peak at 400 nm was still present but now accompanied by a smaller peak corresponding to vesicles of 50–100 nm in diameter, further corroborating the self-division of the fatty acid vesicles. The coherence between confocal microscopy, turbidimetry, and DLS data underscores the robustness of the chemical conversion mechanism in inducing vesicle division.

## Conclusions

We demonstrated a fatty acid–based vesicle division mechanism driven by the chemical potential harvested from the hydration of the condensing agent EDC without relying on complex molecular machinery. Using vesicles comprising a succinic acid derivative (C10) or decanoic acid (DA), we observed that both systems, independently, could undergo division into smaller daughter vesicles at low EDC concentrations by partial conversion of the membrane into oil molecules which, upon hydrolysis bud off new vesicles. This process retained encapsulated contents, suggesting potential protocell-like behavior. The findings indicate that the rate of anhydride hydrolysis and subsequent vesicle formation can be finely controlled by adjusting EDC concentration, offering valuable insights into the dynamics of primitive cellular processes. These insights contribute to the foundational understanding of developing synthetic cells capable of autonomous self-replication. While other methods of dividing vesicles exist, for example, by external feeding of lipids,^7,8,42^ this work is unique in that it uses the chemical potential of a fuel to drive the process. This is, conceptually, closer to how biology uses ATP-driven cell machinery to induce cell division. The vesicle division mechanism observed in this study offers a simplified model compared to other division cycles that often rely on sophisticated lipid bilayer dynamics and external energy sources. However, the simplicity comes at a cost. The division is not self-division but triggered by an external supply of fuel. In the future, such a system should trigger division itself, for example, after growing beyond a certain size. Other limitations should also be addressed in future work. For example, the division acts on existing vesicles and does not produce new amphiphiles. Thus, the vesicles become smaller with each division cycle. In future studies, that can be circumvented by adding both fuel and additional amphiphiles or feeding the vesicles with oil droplets. Finally, because of the simplicity of the division mechanism, it is rather heterogeneous, budding off multiple, different-sized vesicles inward and outward.

In the context of prebiotic chemistry, the relevance of this model lies in its ability to demonstrate a basic form of compartmentalization—an essential feature for the emergence of life. The chemical energy provided by a condensing agent triggers division without requiring mechanical intervention, suggesting that similar processes could have contributed to the formation and evolution of protocell-like structures. Although the system does not couple growth and division, as seen in more complex models, it provides important insights into how primitive membranes might have achieved compartmentalization and division under early Earth conditions. The ability of DA vesicles to undergo EDC-induced division without complete disintegration has significant implications for synthetic biology. This process provides a model for designing robust, self-dividing synthetic cells that mimic early protocellular behaviors. Future research could focus on integrating genetic and metabolic components within these vesicles, advancing our understanding of the minimal requirements for cellular life. The insights gained from this study could inform the development of novel biomimetic materials and systems with applications in drug delivery, biosensing, and the creation of artificial life forms. This detailed analysis enhances our understanding of vesicle dynamics and division, opening new avenues for exploring the origins of life and developing synthetic biological systems.

## Supporting Information Available

Extended materials and methods, supporting tables and figures with additional data and analysis, including rate constants, reaction pH over time, absorbance and titration curves, additional confocal micrographs, and supplementary movie descriptions (PDF)
Movie S1. Morphological transition of a C10 multilamellar vesicle in response to 23 mM EDC at pH 4.9 (MP4)
Time-dependent morphological transition of 65 mM C10 multilamellar vesicles in 200 mM MES buffer at pH 4.94 following the addition of 23 mM EDC (MP4)
Movie S3. Formation and division process of “daughter” vesicles from the surface of a multilamellar vesicle (MP4)
Movie S4. Formation of an elongated “daughter" vesicle from the surface of a multilamellar vesicle (MP4)
Movie S5. Membrane formation on the surface of a C10 multilamellar vesicle in 200 mM MES buffer at pH 4.9 after adding 10 mM EDC (MP4)
Movie S6. Division process of a multilamellar vesicle labeled with 2 μM Nile Red and containing encapsulated material in the presence of 10 mM EDC (MP4)
Movie S7. Division sequence of decanoic acid vesicles at pH 6.8 upon exposure to 0.4 mM EDC (MP4)
