# Supplementary material for: Chemically Driven Division of Protocells by Membrane Budding
Source: J Am Chem Soc. 2024 Nov 27;146(49):33359–67. doi: 10.1021/jacs.4c08226 (PMC11638963; doi:10.1021/jacs.4c08226)
Supplement: Supplementary file 1 — ja4c08226_si_001.pdf [file ja4c08226_si_001.pdf]

Supporting Information for:

Chemically Driven Division of Protocells by Membrane Budding

Authors: Pablo Zambrano[1], Xiaoyao Chen[1], Christine M. E. Kriebisch[1], Brigitte A. K. Kriebisch[1], Oleksii Zozulia[1], Job Boekhoven[1]

Affiliations:

[1]Department of Chemistry, School of Natural Sciences, Technical University of Munich, Lichtenbergstrasse 4, 85748 Garching, Germany.

TABLE OF CONTENTS

Materials 4

Methods 4

Supporting Tables

Table S1: Rate constant used in the kinetic model for asymmetric acid at pH 4.9 fueled with EDC. 16

Table S2: Rate constant used in the kinetic model for symmetric acid at pH 6.8 fueled with EDC. 16

Table S3. Characterization of precursor and product. 16

Table S4. Reaction pH as a function of time for C10. 17

Table S5. Reaction pH as a function of time for decanoic acid. 18

Supporting Figures

Figure S1. C10 phases as a function of pH. 19

Figure S2. Titration curve of 10 mM C10 solution. 20

Figure S3. CVC determination methods for C10 precursor. 21

Figure S4. Confocal micrographs of C10 vesicles in MES buffer. 22

Figure S5. Confocal micrographs of C10 vesicles by anhydride hydrolysis. 23

Figure S6. Confocal micrograph of C10 multilamellar vesicles. 24

Figure S7. Fluorescence Recovery After Photobleaching (FRAP) of C10 multilamellar vesicles. 25

Figure S8. Visualization of Merocyanine-540 interactions with anhydride oil droplets and C10 vesicles. 26

Figure S9. Time-series of C10 multilamellar vesicles after EDC addition. 27

Figure S10. Representative confocal micrographs of C10 vesicles before and after EDC addition. 28

Figure S11. Bright-field microscopy and DLS of C10 vesicle solution before and after EDC addition. 29

Figure S12. Cryo-EM and DLS analysis of C10 vesicles before and after EDC addition. 30

Figure S13. Fluorescence and bright-field microscopy of mixed C10 vesicles post-EDC addition. 31

Figure S14. Confocal and bright-field microscopy of C10 vesicles after EDC addition with agitation. 32

Figure S15. Close-up analysis of C10 vesicles after EDC addition with agitation. 33

Figure S16. Time-series confocal micrographs of C10 vesicle after EDC addition. 34

Figure S17. HPLC data and kinetic model of C10 vesicles with different EDC concentrations. 35
Figure S18. Iterative fueling of C10 multilamellar vesicles with EDC. 36
Figure S19. Confocal micrographs of C10 vesicles with encapsulated labeled-DNA. 37
Figure S20. Collapse of C10 vesicles and release of encapsulated oligo-Atto488. 38
Figure S21. Absorbance vs pH for decanoic acid (DA) and CVC determination. 39
Figure S22. Confocal micrographs of DA at different pH values. 40
Figure S23. Confocal micrographs of DA at different concentrations. 41
Figure S24. Representative confocal micrograph of DA vesicles. 42
Figure S25. Quantification of vesicle counts and diameters after EDC addition to DA vesicles. 43
Figure S26. HPLC data and kinetic model for DA vesicles with different EDC concentrations. 44
Figure S27. HPLC chromatograms showing kinetics of C10 with EDC addition. 45
Figure S28. Mass spectrum of the C10 precursor. 46
Figure S29. 1H-NMR spectrum of the C10 precursor. 47
Figure S30. 13C-NMR spectrum of the C10 precursor. 48
Description of Supplementary Movies 49
References 50

Materials and methods

Materials. We purchased (*E*/*Z*)-2-decen-1-ylsuccinic anhydride (C_{10} anhydride) from TCI Chemicals. Decanoic acid, 1-ethyl-3-(3-dimethylaminopropyl) carbodiimide (EDC), 2-(*N*-morpholino)ethanesulfonic acid (MES) buffer, merocyanine 540 and Nile Red were purchased from Sigma-Aldrich and used without any further purification unless otherwise indicated. DNA-488 (5'-[Atto488]GCCTCTGTGTCGCATCTTCGCGGAGAGTTCCTTT) was purchased from Atto-TEC. HPLC grade acetonitrile (ACN) was purchased from VWR.

Synthesis of the C10 (succinic acid derivative). Five millilitres of 2-decen-1-ylsuccinic anhydride were suspended in 30 millilitres of ultrapure water and stirred for three days. After this period, the reaction mixture was freeze-dried (Lyophylizator: Alpha LDplus, Christ), and the resulting 2-decen-1-ylsuccinic acid (precursor) was stored at -20 °C until further use. We found no starting material by HPLC.

Methods

pH measurements. For pH adjustments of the stock solutions and for measuring pH values, a HANNA HI 2211 pH/ORP Meter equipped with an Ag/AgCl electrode was utilized. Before each use, it was calibrated using standard calibration solutions at pH 7.01 and 4.01.

Mass Spectrometry Analysis. Mass spectrometry analysis was conducted to further characterize the sample. The mass spectra were obtained using an LCMS - c ESI Full ms [50.00-600.00] method in the negative mode. The relative abundances of the detected ions were plotted against their mass-to-charge ratios (m/z).

NMR spectroscopy. The 1H-NMR spectra were recorded on a Bruker AV-400HD NMR spectrometer at 400 MHz or an AV-300HD NMR spectrometer at 300 MHz. All chemical shifts (δ) are reported in parts per million (ppm) relative to the residual proton signal of the solvent. The spectra were analysed using MestreNova software (Version 15.0.1).

^{1}H NMR (400 MHz, DMSO). δ 12.15 (s, 2H), 5.55 – 5.17 (m, 2H), 2.72 – 2.57 (m, 1H), 2.48 – 2.08 (m, 4H), 1.95 (p, J = 6.2 Hz, 2H), 1.37 – 1.15 (m, 10H), 0.90 – 0.80 (m, 3H).

^{13}C NMR (101 MHz, DMSO). δ 175.39, 175.34, 173.09, 173.06, 132.88, 131.99, 126.33, 125.83, 40.71, 40.67, 34.83, 34.74, 34.23, 31.88, 31.26, 29.03, 28.83, 28.76, 28.63, 28.52, 28.43, 26.62, 22.08, 13.93.

Preparation of C10 Vesicles. We employed two methods to obtain vesicles of the appropriate size for monitoring their morphological changes. <u>Method 1:</u> Initially, we dissolve an appropriate amount of lyophilised C10 in chloroform in a 4 mL glass bottle. The chloroform was then evaporated slowly using a gentle stream of nitrogen under a fume hood while rotating the glass bottle for at least 20 minutes. Subsequently, the samples were hydrated with MES buffer 200 mM and the pH was adjusted to the desired value. The bottles were placed on an equatorial rotator for over 24 hours at room temperature. After this period, the vesicles were used immediately. The vesicles are stable for extended periods of time (> 1 week) at room temperature. <u>Method 2:</u> This method consisted of the prolonged hydrolysis of C10 anhydride under vigorous agitation. A suitable volume of anhydride was measured to achieve a final C10 concentration (acid) of 65 mM. The anhydride was placed in a Falcon tube with an adequate MES buffer volume (pH 5.3). The sample was sonicated for 3 minutes. Then, the pH was readjusted as desired. Subsequently, the tube was placed on a Falcon tube shaker (700 osc/min; Flask Shaker, SF1, Cole Parmer ™) for at least 24 hours, regularly checking the pH of the sample. No traces of anhydride were detected after this time using HPLC. The desired final pH to produce C10 vesicles was 4.94.

Encapsulation of labeled DNA-ATTO488 in C10 Vesicles. Two different methods were explored for the encapsulation of labeled DNA. <u>Method 1:</u> As detailed in the **"Preparation of C10 Vesicles"** section, a certain amount of lyophilized C10 was weighed for a final concentration of 100 mM. The precursor mass was dissolved in 50 µL of chloroform. Subsequently, the solvent was carefully evaporated under a gentle stream of nitrogen. Simultaneously, a labelled DNA-ATTO488 solution (final concentration 1.5 µM) was prepared in MES buffer 200 mM and pH 4.9. The C10 film was hydrated with the labeled-DNA solution and stirred overnight. Subsequently, the solution was diluted with MES buffer (pH 4.9) to remove unencapsulated material. <u>Method 2:</u> Additionally, the emulsion-transfer method was adapted[1]. First, an appropriate amount of C10 was weighed for a final concentration of 65 mM. The C10 mass was dissolved in 50 µL of chloroform. Subsequently, the solvent was evaporated using a gentle stream of nitrogen for 15 minutes. Afterwards, the C10 was

resuspended in 500 µL of mineral oil and vortexed or alternatively sonicated for 3 minutes. Then, 500 µL of MES buffer 200 mM pH 4.9 was added to a 1.5 mL Eppendorf tube, and on top of this, 200 µL of the C10-oil mixture was added, left to rest for 30 minutes at room temperature. Meanwhile, a mixture was prepared by adding 200 µL of the C10-oil mixture to a different 1.5 mL Eppendorf tube and adding 5 µL of labelled-DNA 1.5 µM, shaking approximately 15 times vigorously with an Eppendorf tube rack. After 30 minutes, all the content from the second Eppendorf tube (containing labeled DNA-ATTO488) was carefully transferred to the first one and immediately centrifuged at 300 r.c.f. for 10 minutes. Finally, the supernatant (oily phase) was removed, and the vesicles were extracted from the bottom of the Eppendorf tube to be placed directly on an IBIDI µ-slide 15 Well 3D for observation under the microscope.

Preparation of Decanoic Acid (DA) Vesicles. DA vesicle solutions were prepared using a modified method described by Monnard & Deamer [2]. Decanoic acid can form vesicles at a pH close to its apparent pKa, around 7.0 [3]. To achieve the necessary pH, we opted to decrease the pH of a micelle suspension rather than increasing the pH of the pure fatty acid. Initially, a specific amount of DA was weighed and then dissolved in a MES buffer at pH 11, vigorously vortexed. Subsequently, 4 M NaOH was added until the solution became clear, indicating the complete deprotonation of the acid. Finally, the pH was adjusted with 6 M HCl to reach the desired value. The solutions were used immediately after preparation. Nile Red dye (2µM) was used for confocal microscopy experiments.

Reaction cycles. Reaction cycles were started by adding the high-concentration EDC to the acid (vesicles) solution. We carried out all experiments at 21 (±0.5) ºC.

CVC determination. Our study explored different methods to determine the critical vesicle concentration (CVC) for the C10 precursor at a pH of 4.45. Firstly, the Nile Red assay was employed, utilising fluorescence spectroscopy to assess vesicle formation based on the method described by Wanzke et al. [4]. Briefly, different concentrations of a precursor (ranging from 0.01 to 100 mM in 0.2 M MES buffer) were mixed with Nile Red (5 µM) in a 10 mm quartz cuvette (Precision Cells Inc.). Using a Jasco (FP-8300) spectrofluorometer with external temperature control (MCB-100), the fluorescence intensities of the precursor solutions were measured at an emission wavelength of 635

nm and an excitation wavelength of 550 nm. To calculate the corresponding blue shift, the fluorescence intensity of a blank sample (0.2 M MES buffer with 5 μM Nile Red) was subtracted from that of the corresponding test sample. Secondly, we measured the concentration dependence of the 90° scattering intensity using dynamic light scattering (DLS), which detects changes in phase composition as the concentration of C10SA varies, following the methodology outlined by Namani et al. [5]. Thirdly, turbidimetric CVC determinations were conducted, plotting the absorbance at 600 nm as a function of C10SA concentrations using a 200 mM MES buffer, also based on the method by Namani et al [5]. Lastly, we utilized merocyanine 540, a lipophilic probe sensitive to lipid packing, to determine CVC by observing changes in the A565 nm/A525 nm absorbance ratio. For this, 0.5 μL of merocyanine 540 solution was added to a serial dilution of the samples (500 μL) from a 2.5 mM stock solution in ethanol, allowed to equilibrate in the dark for half an hour before measurements, as per the procedures detailed by Williamson et al. [6]. These diverse approaches enabled a comprehensive evaluation of CVC under specified conditions.

Vesicles extrusion (400 nm). A total of 1000 μL of C10 multilamellar vesicles (65 mM) were loaded into a Hamilton syringe (Hamilton™) and carefully placed at one end of the Mini-Extruder (Avanti Polar Lipids), which had been prepared with a 0.1 or 0.4 μm membrane (Nuclepore™ Track-Etched, Whatman™). The extruder parts were pre-wetted to minimize dead volume by passing a syringe filled with MES buffer through the extruder and then discarding the buffer. An empty syringe was positioned at the other end of the Mini-Extruder, ensuring the plunger was set to zero; the syringe automatically filled as the C10 solution was extruded through the membrane. Nine repetitive steps were used to achieve reproducible and optimal results (100 and 400 nm).

pKa determination / pH titration. For the titration of a 10 mM C10 concentration, the necessary amount of C10 for a 200 μL total volume was first suspended in 100 μL of MQ-water. To achieve a pH greater than 10, 90 μL of 0.05 M NaOH was added to the aqueous C10 solution. The volume was then adjusted to 200 μL, and the samples were vortexed to ensure the C10 dissolved completely. The titration was conducted at 25 °C by gradually adding small volumes of 0.1 M HCl. After each HCl addition, the samples were vortexed again. The pH levels were recorded using a HI 2210 pH meter

by HANNA® Instruments. The titration curves were subsequently analyzed using the Hyperquad2008 software[7] to determine the apparent pKa of the precursor.

Cryo-TEM experiments. The grids were prepared as follows: Cu-grids (C-flat™ 2/1 on 400 copper mesh) were glow-discharged for 90 seconds at 45 mA and 3×10^{-2} mbar. Subsequently, 5 µL of the sample (as prepared above) was applied to the Cu-grids and then plunge-frozen using a Vitrobot Mark IV (FEI, now Thermo Scientific) under the specified conditions: 100 % humidity, a temperature of 22 °C, a 30 sec wait time, a blot force of -1, and a blot time of 2.5 sec. The cryo-TEM grids were then stored in liquid nitrogen until they were ready for imaging on the Tecnai Spirit microscope (FEI/Thermo Fisher). The cryo-TEM grids were inserted into a Gatan cryo-transfer-specimen holder for imaging. Images were captured using a TVIPS-416 CCD camera (TVIPS) with serialEM software.

Dynamic Light Scattering (DLS). DLS analysis was conducted using a Litesizer 500 particle size analyzer from Anton Paar. The instrument is fitted with a 40 mW, 658 nm laser diode. Samples were placed into the instrument using a 50 µL quartz cuvette (3x3 mm light path, Hellma Analytics).

HPLC. We used a ThermoFisher Vanquish Duo UHPLC, a Hypersil Gold 100 x 2.1 mm C18 column (3 µm pore size) to monitor the concentration profiles of each reactant of the chemical reaction network. We prepared 400 uL samples according to the sample preparation protocol described above into a screw cap HPLC vial. Samples were injected directly from the HPLC vial without any further dilution. We injected 2.5 uL to detect the C10 acid, anhydride, and EDC. We used a UV/Vis detector at 220 nm for the detection. A linear gradient of MQ water: ACN with 0.1 % TFA was used to separate the compounds. We used a linear gradient from 60:40 to 2:98 in 5 minutes, followed by 1 minute at 2:98 for the separation, 0.2 min to 60:40, and 3.2 min equilibrium at 60:40. We performed calibration curves of the compounds in triplicates. Calibration values and retention times are given in Table S4.

UV/Vis Spectroscopy. Absorbance measurements using UV-Vis at a wavelength of 600 nm were conducted using a SpectraMax® ABS Plus plate reader from Molecular Devices. The samples were set up in 96 well plates, using a volume of 100 µL. Turbidity development was tracked following the addition and thorough mixing of the carbodiimide fuel EDC by pipetting. All measurements were performed in triplicate.

Fluorescence confocal microscopy of C10 and decanoic acid vesicles. We used a Leica TCS SP8 confocal microscope with a 63x oil immersion objective to image vesicles. Vesicle samples were prepared as described above. All experiments used 20 µL of sample in an IBIDI µ-slide 15 Well 3D. Nile Red dye at a concentration of 2 µM was used in all experiments to label the membrane of the vesicles, and they were excited with a 561 nm laser and imaged at 570-700 nm. On the other hand, in the encapsulation experiments, dual-laser imaging was used: a 488 nm laser for excitation with images between 500 and 550 nm to visualize ATTO488-labeled DNA and a 561 nm laser for excitation with images between 605 and 750 nm to visualise Nile Red-labeled membranes. Images were captured at a resolution of 1024 × 1024 over an 184 × 184 µm area or 2048 × 2048 over an 246.03 × 243.03 µm area. For time series, images were obtained at various time points to track morphological changes in the vesicles (Scan mode xyzt; Resolution of 1024 × 1024 over an 184 × 184 µm area; Speed 600 Hz). The reaction cycles of C10 and DA vesicles with EDC were initiated in an Eppendorf tube and quickly transferred the sample (20 µL) to the µ-slide. Experiments were also performed by directly injecting the fuel into the µ-slide containing vesicles in solution. All acquired images were processed and analyzed using ImageJ software.

Fluorescence Recovery After Photobleaching (FRAP) experiments were conducted using a Leica TCS SP8 confocal microscope equipped with a 63x water immersion objective. The samples, composed of multilamellar vesicles of C10 (65 mM in MES 200 mM at pH 4.94), were prepared in Ibidi micro-well chambers with a total sample volume of 20 µL. For fluorescence labelling, the vesicle membranes were stained with Merocyanine-540 (MC540). A 561 nm laser was used for excitation, while the images were collected between 605 and 750 nm to visualize the MC540-labeled membranes. The photobleaching region of interest (ROI) was defined as 2 µm x 2 µm. A sequence of 20 pre-bleach images was acquired, followed by the photobleaching event. After the bleach, fluorescence recovery was monitored over a period during which 800 images were captured in total. The raw fluorescence data was processed using ImageJ, with double normalization applied to account for intensity fluctuations following established protocols from the literature. The fluorescence recovery data was fitted to a first-order exponential equation to extract the diffusion coefficient and half-time recovery of the vesicles.

Confocal micrographs acquisition for quantification. We quantified the production of daughter vesicles using confocal microscopy. Initially, the control sample (C10 vesicles, 65 mM) was placed in an Ibidi Chamber (20 µL of sample) and stabilized for 60 minutes before imaging. The images were acquired with a resolution of 2048 × 2048 pixels at a scanning speed of 100 Hz, covering an area of 246.03 × 246.03 µm. To quantify the number of GUVs, we captured confocal micrographs by selecting the equatorial plane where the maximum number of GUVs were visible and in focus. This approach minimized artifacts arising from sample movement and focus variations during multiple z-plane imaging, ensuring consistent and reproducible measurements[8–12]. Before imaging, the GUVs were allowed to settle on the coverslip for 60 minutes. Subsequently, EDC was added to the samples at various concentrations (16, 23, 32, and 65 mM) in separate Eppendorf tubes. The samples were gently mixed to ensure uniform distribution of the EDC and placed in Ibidi Chamber (20 µL of sample). The GUVs were allowed to settle on the coverslip for 60 minutes. The imaging adquisition procedure was then repeated after 1.5 hours of EDC adding. A total of 25 confocal images were taken for each EDC concentration and analyzed using ImageJ software.

Vesicle quantification using ImageJ. To quantify vesicles using Fiji (ImageJ), we acquired confocal microscope images with a resolution of 2048 × 2048 pixels at a scanning speed of 100 Hz, covering an area of 246.03 × 246.03 µm. The images were converted to grayscale, and contrast was enhanced. Background subtraction was applied to address any unevenness. We used thresholding to distinguish vesicles from the background, selecting an appropriate method like Otsu or Yen, and adjusting threshold levels until vesicles were clearly defined. Vesicles were analyzed by setting a particle size range, typically between 0.5–50 µm², to exclude noise and artifacts. Results were displayed, summarized, and visually represented with outlines. We use Prism 9 for macOS (version 9.5.0) to perform the statistical analysis and plot the results.

Statistical. All statistical analyses were performed using GraphPad Prism version 9.5.0(525), with a significance level set at $\alpha=0.05$. Data are presented as mean ± standard deviation (SD) unless otherwise stated, and error bars in the figures represent SD. The one-way analysis of variance (ANOVA) was used to determine significant differences between multiple groups, specifically the number of vesicles

generated after adding different EDC concentrations. To confirm the assumptions of homogeneity of variances, the Brown-Forsythe and Bartlett's tests were conducted, yielding p<0.0001****, thereby confirming variance homogeneity. An unpaired t-test was employed to compare vesicle diameters before and after adding 65 mM EDC. The sample sizes were n=68 for the 'Before fuel' group and n=8131 for the 'EDC 65 mM' group. Normality tests were performed to assess the distribution of vesicle sizes, including the D'Agostino & Pearson, Anderson-Darling, Shapiro-Wilk, and Kolmogorov-Smirnov tests. These tests evaluate whether the data follow a normal distribution, a prerequisite for certain parametric statistical tests. The control group ('Before fuel') passed the D'Agostino & Pearson test ($p = 0.0713$), suggesting a normal distribution. However, it failed the Anderson-Darling ($p = 0.0032$), Shapiro-Wilk ($p = 0.0028$), and Kolmogorov-Smirnov ($p = 0.0059$) tests, indicating some deviations from normality. In contrast, the 'EDC 65 mM' group showed significant deviations from normality across all tests ($p < 0.0001$ for each test). For the graphical representation in Figure E, bars represent vesicle size histograms for the 'Before fuel' group (n = 68, blue) and '65 mM EDC' group (n = 8131, orange). Lines represent Gaussian fits: 'Before fuel' group (blue) with amplitude 0.1236, mean 15.36 μm, and SD 6.820 μm; '65 mM EDC' group (orange) with amplitude 0.5916, mean 2.684 μm, and SD 1.103 μm. The same statistical procedures were applied to decanoic acid reaction cycles.

Kinetic Model for fuel EDC

The kinetic model was devised in Python (Table S1), as described by Chen *et al.*[1] and Hartly's work.[2] The kinetic model calculates the concentration of all relevant components over time in the reaction cycle based on four differential equations, which describe five chemical reactions (*vide infra*): 1) direct hydrolysis of fuel (EDC) (r_0), 2) the activation of acid to the intermediate O-acylisourea (r_1), 3) the spontaneous hydrolysis of O-acylisourea (r_2), 4) the formation of anhydride *via* the intramolecular reaction of intermediate O-acylisourea with the second carboxylate of the acid (r_3), and 5) the spontaneous hydrolysis of anhydride (r_4) and the intramolecular rearrangement of the O-acylisourea to the N-acylisourea.

All parameters (CVC, S, S_{eff}, and k_{cat}) and k-values (k_1, k_a, k_4) were free parameters to optimize. To minimize the set of free parameters, we experimentally determined the Critical

Vesicle Concentration (CVC) and monitored the background hydrolysis of fuel in buffer (k_0). We fitted those remaining parameters and kinetic rate constants as described below. All the rate constant values are listed in Table S1-2.

Reaction 0 (k_0):

EDC hydrolyzes directly with a first-order rate constant of 0.0042 min^{-1} at pH 4.9, as determined by HPLC.

Scheme S1: *Kinetic model – Reaction 0 (k_0).*

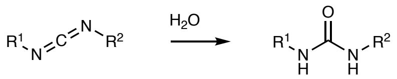

Reaction 1 (k_1):

Fuel activates acid to its corresponding O-acylisourea in a second-order reaction.

Scheme S2: *Kinetic model – Reaction 1 (k_1).*

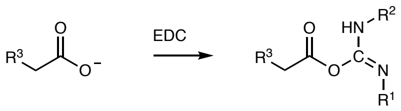

Reaction 2 (k_2):

The O-acylisourea spontaneously hydrolyzes in a pseudo-first-order reaction.

Scheme S2: *Kinetic model – Reaction 2 (k_2).*

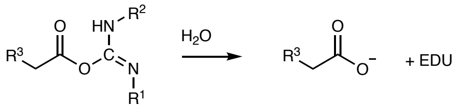

Reaction 3 (k_3):

O-acylisourea reacts with the second carboxylate of the acid to form the intramolecular anhydride.

Scheme S3: *Kinetic model – Reaction 3 (k_3).*

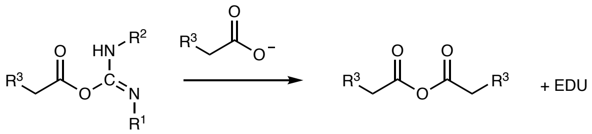

Reaction 4 (k_4):

The anhydride spontaneously hydrolyzes in a pseudo-first-order reaction.

Scheme S4: *Kinetic model – Reaction 4 (k_4).*

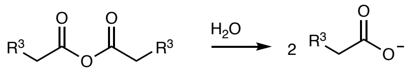

Reaction 5 (k_5):

The O-acylisourea rearranges to N-acylisourea. As N-acylisourea was not observed, its rate constant was set to 0.

Scheme S5: *Kinetic model – Reaction 5 (k_5).*

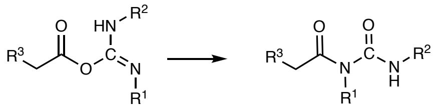

Ordinary differential equations (ODEs) for asymmetric anhydride

The following set of ODEs was used to describe the systems. A steady-state approximation was applied to remove any explicit dependence on the concentration of O-acylureas. We updated the Python script described in Chen *et al.*,[1] for the set of differential equations described below to fit the experimental data. Ac, refers to acid, An refers to anhydride, F refers to fuel, W to waste. We used the ratios $k_a = k_2/k_3$. As the O-acylisourea was not observed, the individual values of k_2, and k_3 cannot be accurately extracted. Instead, we used a steady state approximation as reported previously, using the ratios $k_a = k_2/k_3$.[1-4] CVC is the critical vesicle concentration, S is the anhydride solubility, S_{eff} is the effective solubility of anhydride, and k_{cat} is the solubilization capacity.

We implemented the described self-amplifying decay as stated below, and described by Schwarz *et al.*[5] using equations 1-10. The effective solubility of the anhydride S_{eff}, describes that anhydride is more soluble inside of acid-based vesicles. Thus, S_{eff} is a constant below the CVC of the acid but increases with increasing precursor concentration above the CVC of the acid. The solubilization capacity k_{cat} describes how effectively the surfactant precursor increases the solubility of the anhydride in dependence on the number of precursor molecules above the CVC. We applied two differential equations to describe the anhydride hydrolysis

rate above and below its solubility S. Above its solubility S, the anhydride hydrolysis rate is constant as long as S_{eff} is constant (eq. 1). Below its solubility S, the anhydride hydrolysis rate is a first-order rate depending on the anhydride concentration eq. 2).

If $[Ac]$ < CVC:

$$S_{eff} = S \quad \text{eq. 1}$$

Else:

$$S_{eff} = S + k_{cat} * ([Ac] - CVC) \quad \text{eq. 2}$$

If $[An]$< S_{eff}:

$$\frac{d[F]}{dt} = -k_0 * [F] - k_1 * [F] * [Ac] \quad \text{eq. 3}$$

$$\frac{d[Ac]}{dt} = -k_1 * [F] * [Ac] + k_4 * [An] + \frac{k_1 * k_a * [Ac] * [F]}{k_a + 1} \quad \text{eq. 4}$$

$$\frac{d[An]}{dt} = +\frac{k_1 * [Ac] * [F]}{k_a + 1} - k_4 * [An] \quad \text{eq. 5}$$

$$\frac{d[W]}{dt} = +k_0 * [F] + \frac{k_1 * [Ac] * [F]}{k_a + 1} \quad \text{eq. 6}$$

Else:

$$\frac{d[F]}{dt} = -k_0 * [F] - k_1 * [F] * [Ac] \quad \text{eq.7}$$

$$\frac{d[Ac]}{dt} = -k_1 * [F] * [Ac] + k_4 * (\mathrm{S} + \mathrm{kcat} * ([Ac] - \mathrm{CVC})) + \frac{k_1 * k_a * [Ac] * [F]}{k_a + 1} \quad \text{eq. 8}$$

$$\frac{d[An]}{dt} = +\frac{k_1 * [Ac] * [F]}{k_a + 1} - k_4 * (\mathrm{S} + \mathrm{kcat} * ([Ac] - \mathrm{CVC})) \quad \text{eq. 9}$$

$$\frac{d[W]}{dt} = +k_0 * [F] + \frac{k_1 * [Ac] * [F]}{k_a + 1} \quad \text{eq. 10}$$

Ordinary differential equations (ODEs) for symmetric anhydride

The following set of ODEs was used to describe the systems. We updated the Python script described in Chen *et al.*[1] for the set of differential equations described below to fit the experimental data by implementing the described self-amplifying decay as stated below and

described by Schwarz *et al.*[5] using equations 11-21. Ac, refers to acid, An refers to anhydride, F refers to fuel, N to N-acylisourea, W to waste.

If $[Ac]$ < CVC:

$S_{eff} = S$ eq. 11

Else:

$S_{eff} = S + k_{cat} * ([Ac] - CVC)$ eq. 12

If $[An]$< S_{eff}:

$$\frac{d[F]}{dt} = -k_0 * [F] - k_1 * [F] * [Ac] \quad \text{eq. 13}$$

$$\frac{d[Ac]}{dt} = -k_1 * [F] * [Ac] + 2 * k_4 * [An] + \frac{k_1 * k_3 * [Ac] * [F]}{k_3 + k_5 + k_2 * [Ac]} - \frac{k_1 * k_2 * [Ac] * [F]}{k_3 + k_5 + k_2 * [Ac]} \quad \text{eq. 14}$$

$$\frac{d[An]}{dt} = +\frac{k_1 * k_2 * [Ac] * [Ac] * [F]}{k_3 + k_5 + k_2 * [Ac]} - k_4 * [An] \quad \text{eq. 15}$$

$$\frac{d[N]}{dt} = +\frac{k_1 * k_5 * [Ac] * [F]}{k_3 + k_5 + k_2 * [Ac]} \quad \text{eq. 16}$$

$$\frac{d[W]}{dt} = +k_0 * [F] + \frac{(k_2 * [Ac] + k_3) * k_1 * [Ac] * [F]}{k_2 + k_3 + k_2 * [Ac]} \quad \text{eq. 17}$$

Else:

$$\frac{d[F]}{dt} = -k_0 * [F] - k_1 * [F] * [Ac] \quad \text{eq. 18}$$

$$\frac{d[Ac]}{dt} = -k_1 * [F] * [Ac] + 2 * k_4 * (\mathrm{S} + \mathrm{kcat} * ([Ac] - \mathrm{CVC})) + \frac{k_1 * k_3 * [Ac] * [F]}{k_3 + k_5 + k_2 * [Ac]} - \frac{k_1 * k_2 * [Ac] * [F]}{k_3 + k_5 + k_2 * [Ac]} \quad \text{eq. 19}$$

$$\frac{d[An]}{dt} = +\frac{k_1 * k_2 * [Ac] * [Ac] * [F]}{k_3 + k_5 + k_2 * [Ac]} - k_4 * (\mathrm{S} + \mathrm{kcat} * ([Ac] - \mathrm{CVC})) \quad \text{eq. 20}$$

$$\frac{d[W]}{dt} = +k_0 * [F] + \frac{(k_2 * [Ac] + k_3) * k_1 * [Ac] * [F]}{k_2 + k_3 + k_2 * [Ac]} \quad \text{eq. 21}$$

Python codes.

We used the previously described Python script from *Chen et al.*[1] to fit the experimental data and update it for our set of ODEs. The code solves the ODE system and fits it to the experimental data, minimizing an error function, using the lmfit package (https://zenodo.org/record/11813). We further minimized the error of the kinetic constants, performing a bootstrap with 5 iterations in the fitting that generates a distribution of kinetic constants. For the fitting, we used the median of the distribution to be more robust towards outliers compared to the mean. We used the rootsquared mean error of the fitting to calculate the 95 % confidence interval. We evaluated the goodness of fitting by calculating the R2 error.

We obtained the kinetic constants by fitting the kinetic profiles of fuel, acid, anhydride, and waste for one set of typical reaction conditions. We validated the obtained kinetic constants by predicting the data set at different fuel levels. The set of rate constants fitted those reasonably well (Table S1 and S2).

Table S1: Rate constant used in the kinetic model for asymmetric acid at pH 4.9 fueled with EDC.

	K_0 (min^{-1})	k_1 ($mMmin^{-1}$)	k_a	k_4 (min^{-1})	CVC (mM)	S (mM)	S_{eff}	k_{cat}	Half-life* (min)
C10 acid	0.0043	0.173	0.0058	0.319	0.96	0.11	0.074	0.074	2.17

* Half-life of anhydride, calculated by ln(2)/k_4.

Table S2: Rate constant used in the kinetic model for symmetric acid at pH 4.9 fueled with EDC.

	K_0 (min^{-1})	k_1 ($mMmin^{-1}$)	k_2 ($mM\,min^{-1}$)	k_3 (min^{-1})	k_4 (min^{-1})	CVC (mM)	S (mM)	S_{eff}	k_{cat}	Half-life* (min)
Decanoic acid	0.0043	0.023	0.096	0.1	0.5	40	0.01	0.1	0.1	1.39

* Half-life of anhydride, calculated by ln(2)/k_4.

Supporting Tables

Table S4. Characterization of precursor and product.

Name	Structure	Mass [g/mol] Calc.	Mass [g/mol] Obs.	Wavelength (detection)	Retention time [min] @ 220 nm	Calibration value (mAU/mM)
**C10 acid**	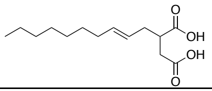	256.17 $C_{14}H_{24}O_4$	255.32 [M-H]-	220 nm	6.237	1.7702
**C10 anhydride**	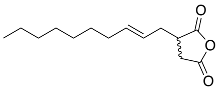	238.33 $C_{14}H_{22}O_3$	-	220 nm	4.807	0.6807

Table S5. Reaction pH as a function of time for C10 (65 mM; MES buffer 200 mM) with EDC 23 mM.

Reaction Time [min]	pH
0	4.94
5	4.98
10	5.01
15	4.96
20	4.91
30	4.91
40	4.91
60	4.92

Table S6. Reaction pH as a function of time for decanoic acid (50 mM) with EDC 0.4 mM.

**Reaction time [min]**	**pH**
0	6.74
5	6.81
10	6.76
15	6.75
20	6.75
30	6.77
40	6.76
60	6.77

Supporting Figures.

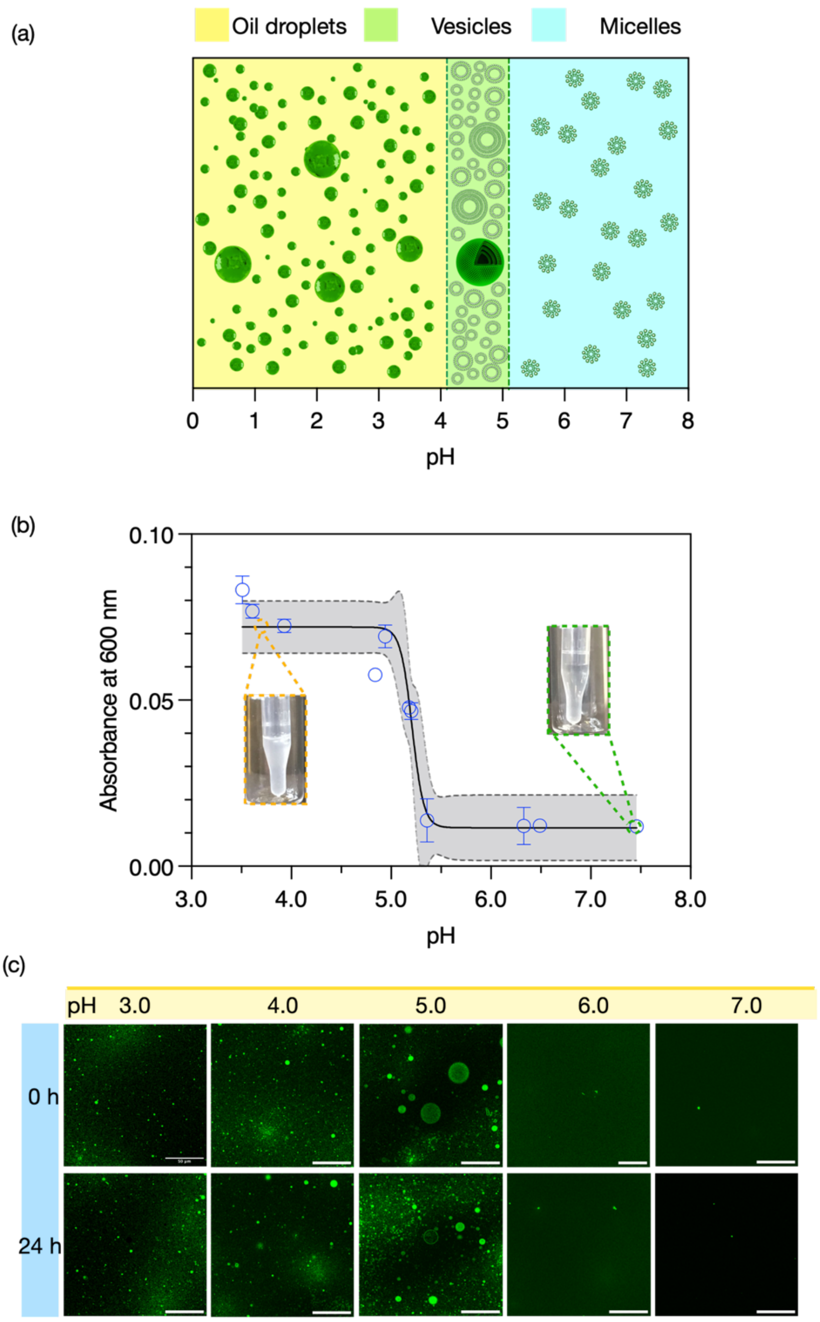

Figure S1. (a) Representative schematic of the oil droplet, vesicle (membranous), and micelle phases for C10 as a function of pH. (b) Plot of absorbance at 600 nm of C10 65 mM in MES buffer vs pH. Vesicles are present at values close to the inflection point of the curve. Error bars are standard deviations (SD) with n = 3 (c) Confocal micrographs of C10 vesicles (20 mM) in MES buffer (200 mM) at different pH and incubation times at room temperature. Dye: Nile red 2 μM. Scale bar 50 μm.

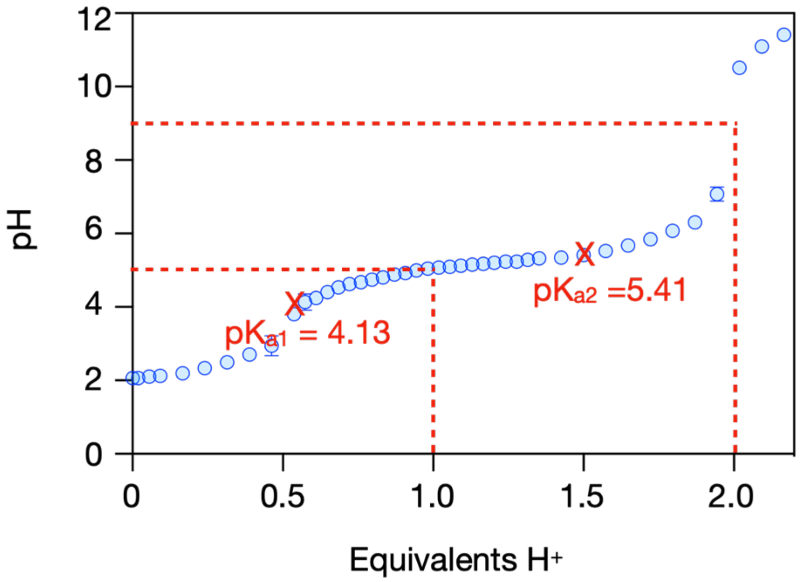

Figure S2. Titration curve of a 10 mM C10 solution, adjusted with 0.05 M NaOH and titrated with 0.1 M HCl at room temperature. The curve represents the apparent pKa values of the system. Error bars represent standard deviations (SD), with n = 3.

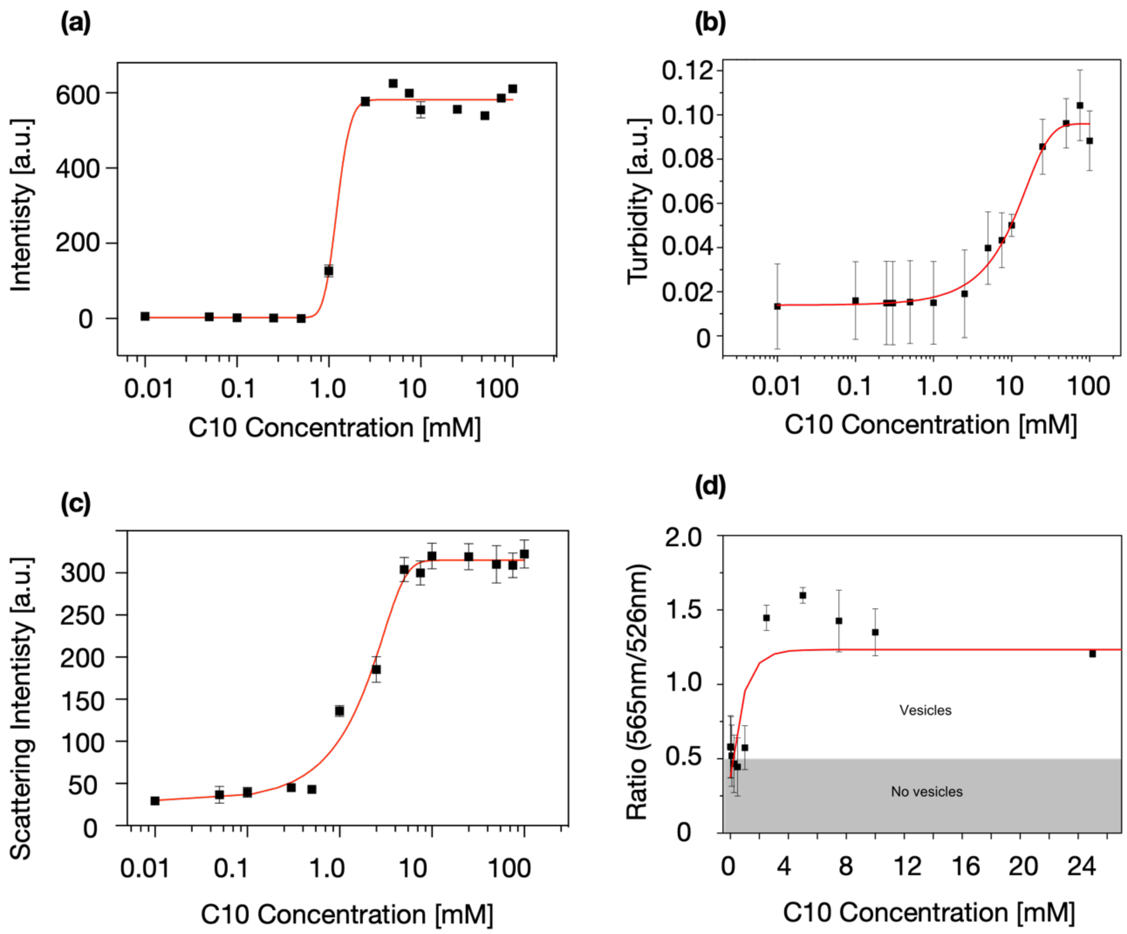

Figure S3. CVC determination methods for C10 precursor: (a) Nile Red fluorescence assay (CVC =1 mM); (b) Turbidimetric CVC determinations plotted against C10 concentrations based on absorbance at 600 nm (CVC = 1.5 mM); (c) Concentration-dependent 90° DLS scattering intensity (CVC 1 mM); (d) CVC determination using merocyanine 540 as a solvatochromic probe, monitoring lipid packing in vesicles through A565 nm/A525 nm absorbance ratio changes (CVC = 1.5 mM). All assays utilized a 200 mM MES buffer. Error bars are standard deviations (SD) with n = 3.

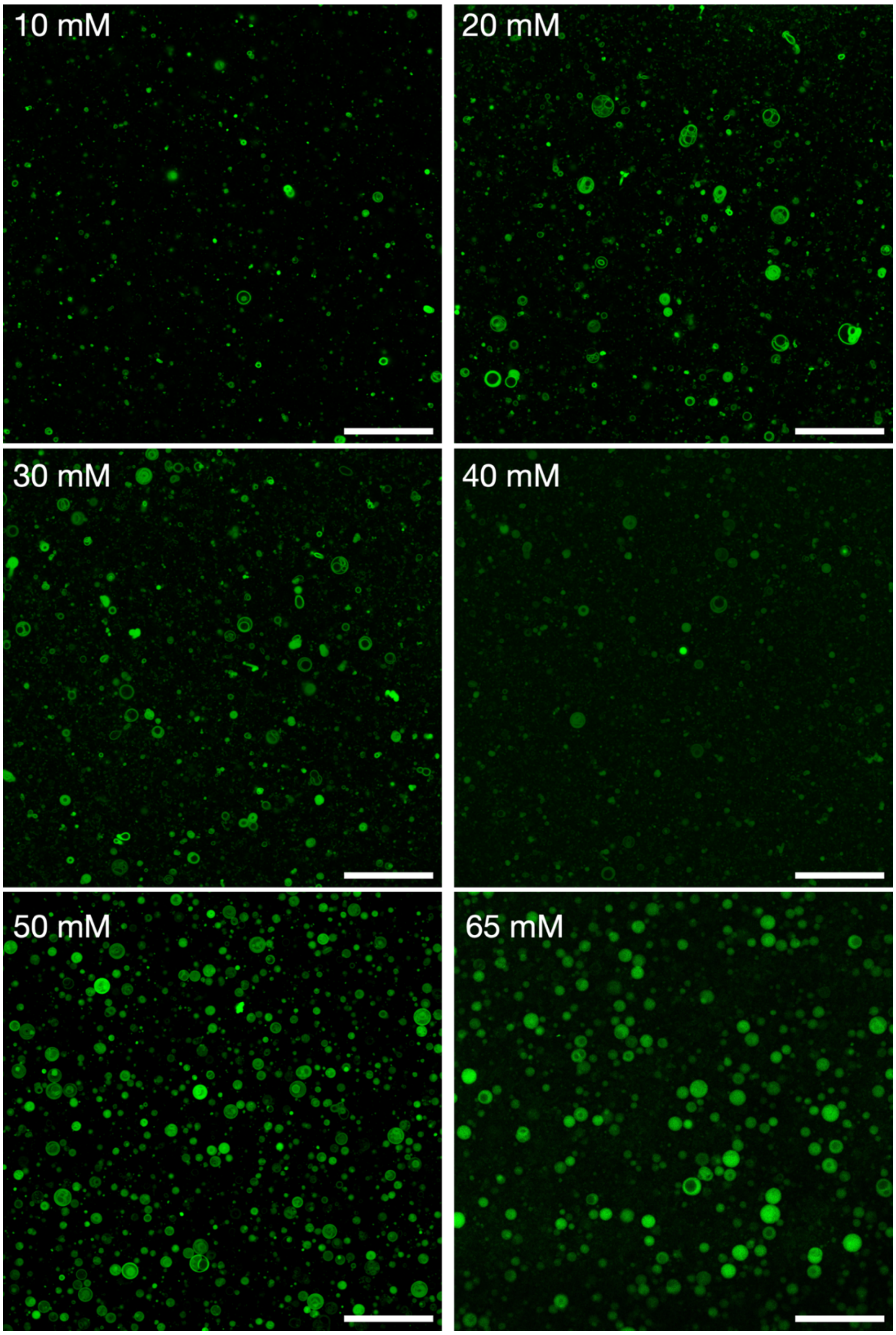

Figure S4. Confocal micrographs of C10 vesicles in MES buffer 200 mM and pH 4.94, obtained by gentle hydration of thin film starting from lyophilized acid. Dye: Nile Red 2 μM. Scale bar: 20 μm.

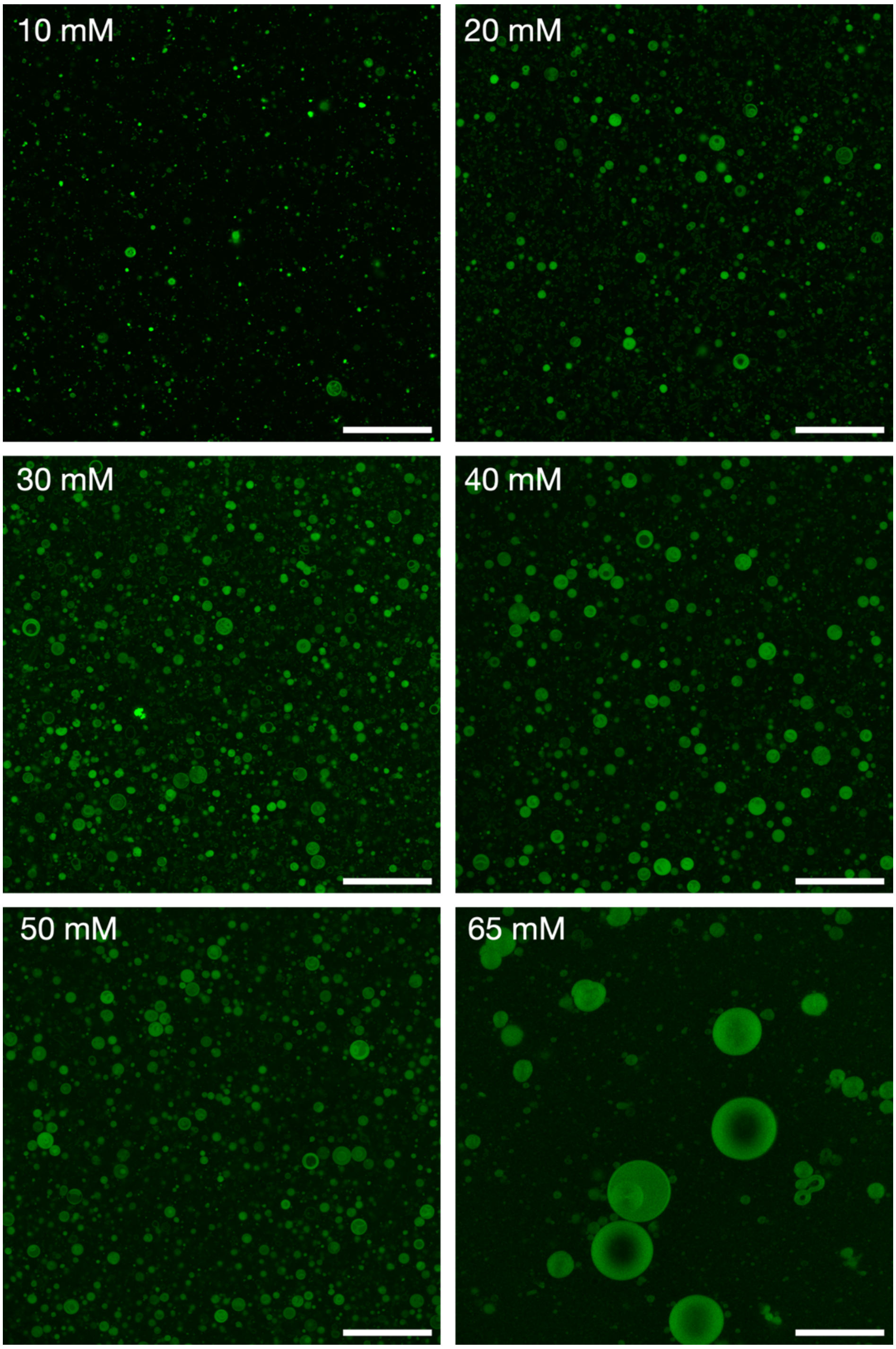

Figure S5. Confocal micrographs of C10 vesicles in 200 mM MES buffer at pH 4.94, obtained by hydration of anhydride and agitation for > 24 h at room temperature. Dye: Nile red 2 μM. Scale bar: 20 μm.

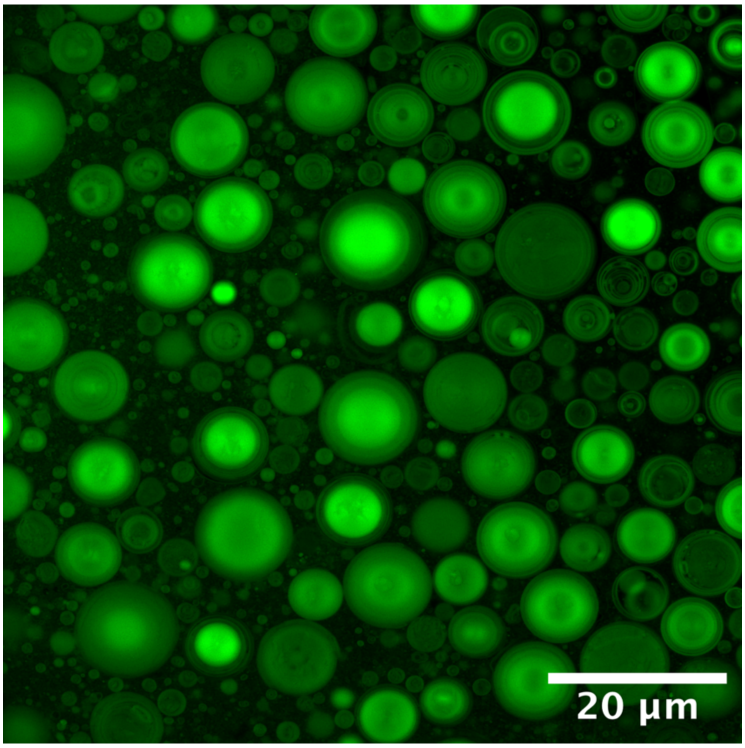

Figure S6. Confocal micrograph of multilamellar vesicles of C10 65 mM in MES 200 mM, pH 4.93, obtained by anhydride hydrolysis method after 28 h of vigorous vortexing (see ***Preparation of C10 vesicles*** section). Dye: Nile Red 20 µM.

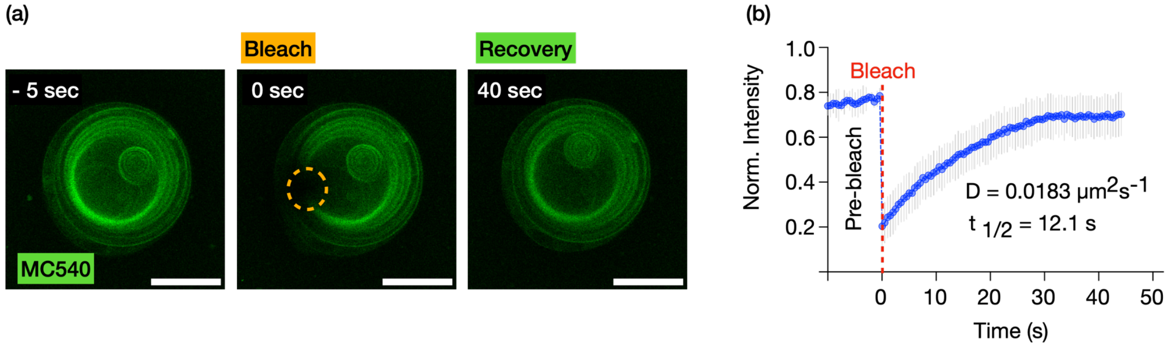

Figure S7. Fluorescence Recovery After Photobleaching (FRAP) analysis of MC540-labeled multilamellar vesicles from three independent experiments. **(a)** Representative fluorescence images of vesicles labeled with Merocyanine-540 (MC540) before bleaching (-5 sec), immediately after photobleaching (0 sec), and during the recovery phase (40 sec). The region of interest (dotted circle) was photobleached, and fluorescence recovery was tracked over time. Scale bars represent 5 μm. **(b)** Normalized fluorescence intensity curve from three independent FRAP experiments showing the average recovery of fluorescence over time after photobleaching. The recovery curve was fitted to a first-order exponential equation, yielding a diffusion coefficient (D) of 0.0183 $\mu m^2/s$ and a half-time recovery ($t_1/_2$) of 12.1 s. Error bars represent the standard deviation from the mean fluorescence intensity across the three experiments.

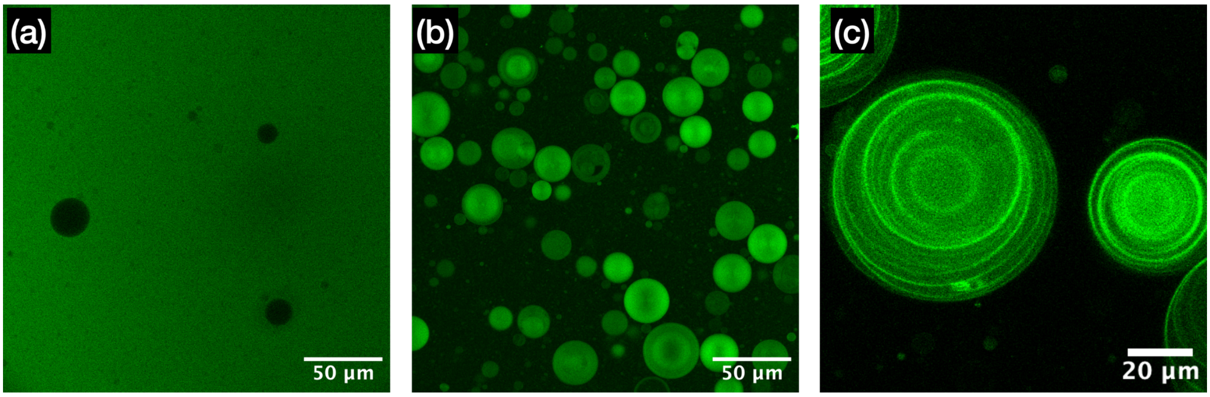

Figure S8: Visualization of Merocyanine-540 interactions with anhydride oil droplets or C10 vesicles. **(a)** Anhydride oil droplets in 200 mM MES buffer at pH 4.94 were imaged 10 minutes after incubation without vigorous dispersion (no ultrasonication). Merocyanine-540 (2 µM) was added to evaluate its affinity for the oil droplets, but no interaction was observed, as shown by the lack of fluorescence. **(b)** Vesicles formed from 65 mM C10 acid, prepared by vigorous dispersion of the anhydride over 28 hours, showing clear labeling with Merocyanine-540. **(c)** A magnified view of the vesicles reveals additional structural details of the multilamellar organization.

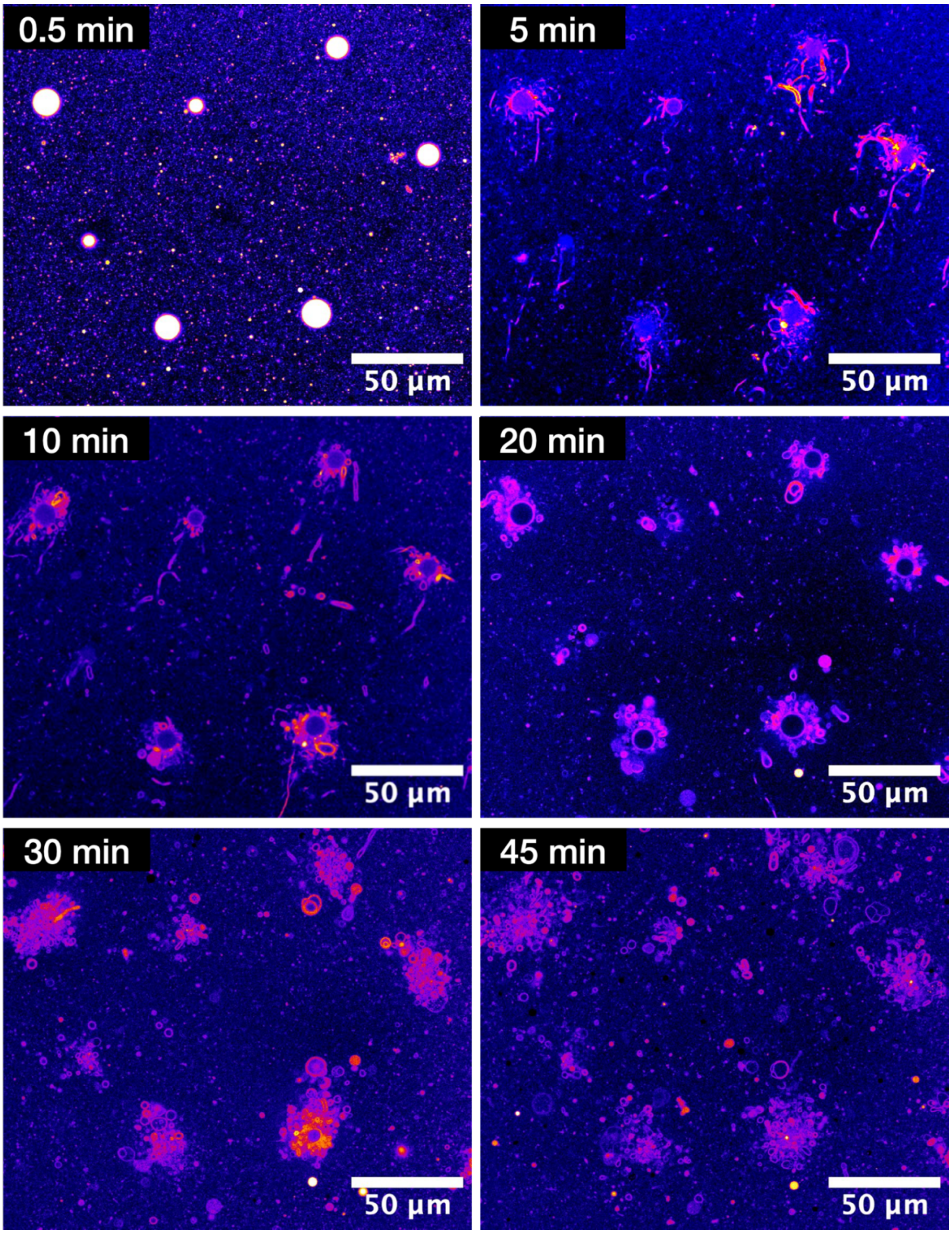

Figure S9. Confocal micrographs of time-dependent morphological transition of 65 mM C10 multilamellar vesicles in 200 mM MES and pH 4.94 after addition of 23 mM EDC. Vesicles were stained with Nile Red 2 µM. Images obtained from Movie S5.

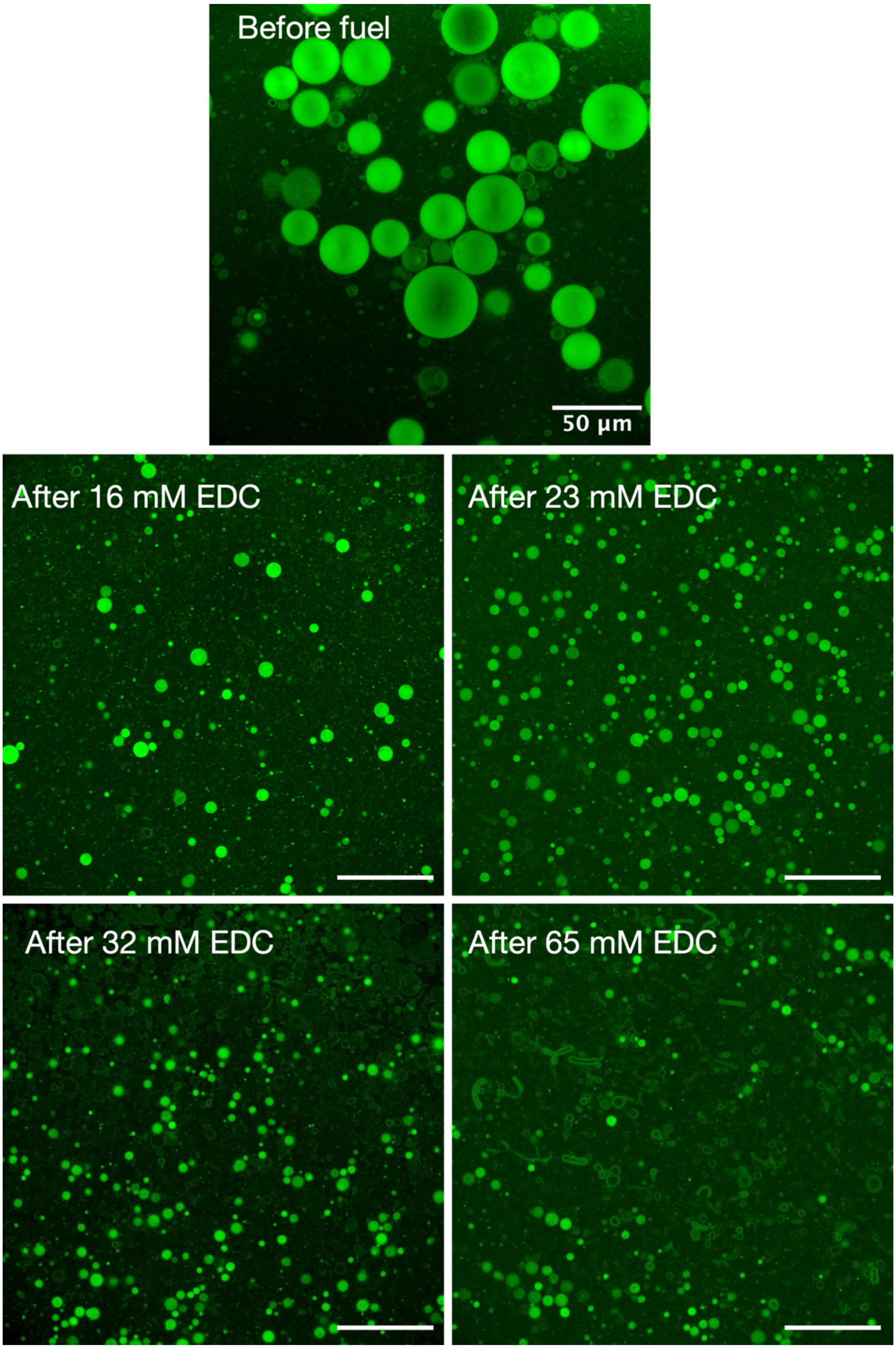

Figure S10. Representative confocal micrographs of C10 vesicles (65 mM, 200 mM MES, pH 4.94), before and after 1.5 hours of EDC addition at different concentrations. Dye: Nile red 2 µM. Scale bar: 20 µm.

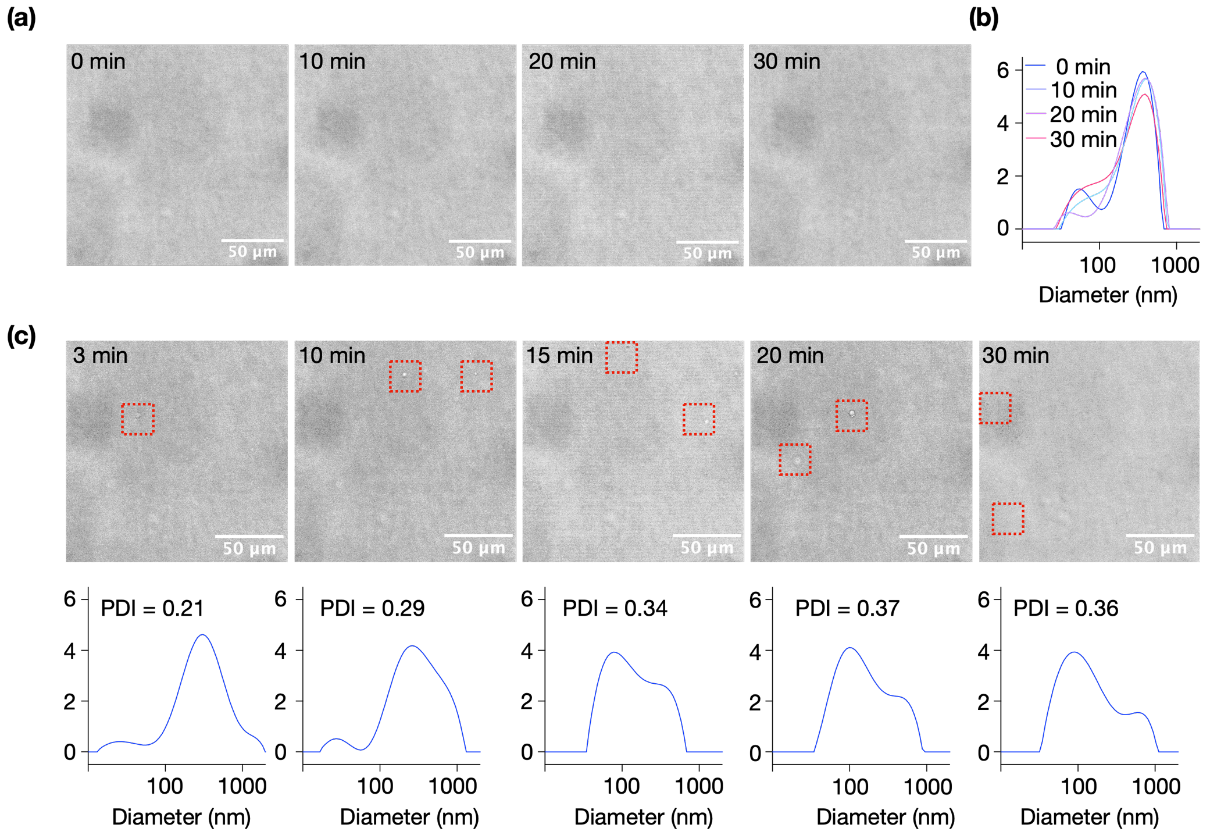

Fig. S11. (a) Bright-field microscopy images of a 65 mM C10 vesicle solution obtained by extrusion through a 400 nm membrane in 200 mM MES buffer at pH 4.94. The vesicles were monitored to investigate potential fusion before adding EDC; (b) Dynamic Light Scattering (DLS) data for the same vesicle solution at different time points. The polydispersity indices were as follows: 0 min = 0.29; 10 min = 0.31; 20 min = 0.30; and 30 min = 0.32. (c) Bright-field microscopy images of a 65 mM C10 vesicle solution obtained by extrusion through a 400 nm membrane in 200 mM MES buffer at pH 4.94 at different times after adding 23 mM EDC. Red frames indicate the presence of droplets generated by the fusion of smaller droplets. Below each image, corresponding DLS data is shown (10 runs each).

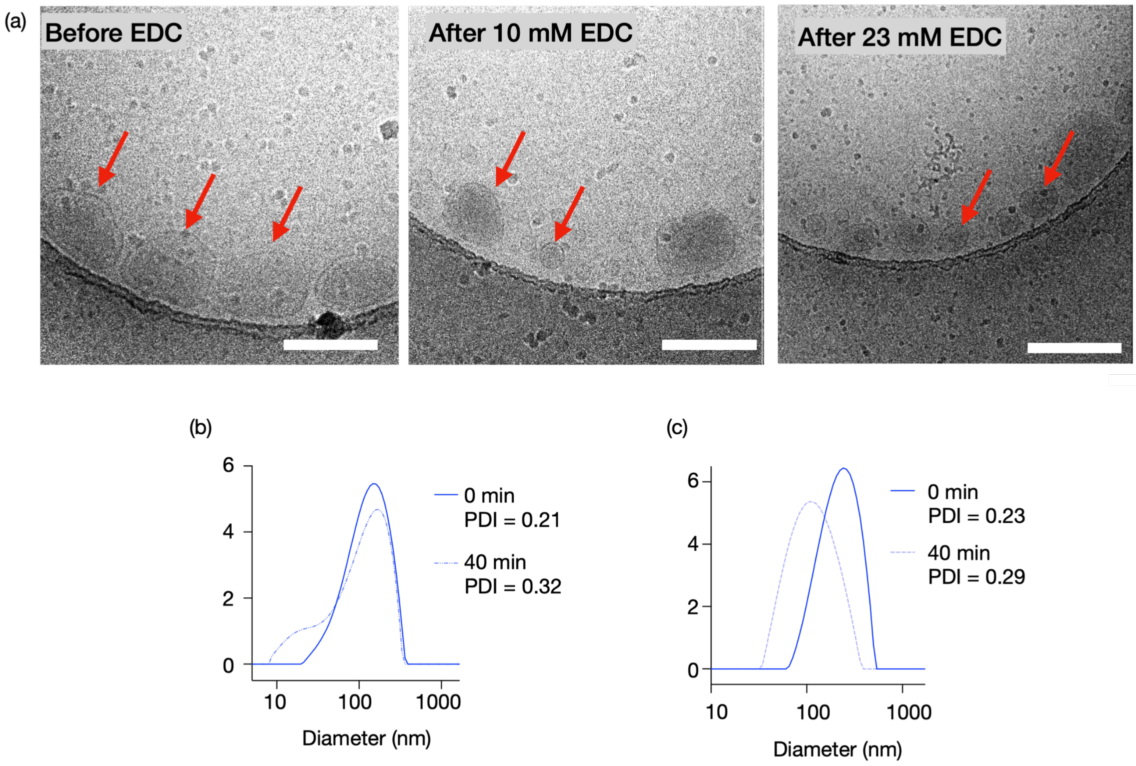

Figure S12: Cryo-EM images and DLS analysis of extruded C10 vesicles before and after the addition of EDC. (a) Cryo-EM images of C10 vesicles (65 mM, pH 4.94, in 200 mM MES buffer) extruded through a 100 nm membrane. Images were taken before the addition of EDC, after the addition of 10 mM EDC, and after the addition of 23 mM EDC. Red arrows indicate the vesicle membranes. Scale bars = 200 nm. (b, c) Dynamic Light Scattering (DLS) data corresponding to vesicles after 10 mM EDC addition (b) and after 23 mM EDC addition (c), showing the size distribution of vesicles at each condition.

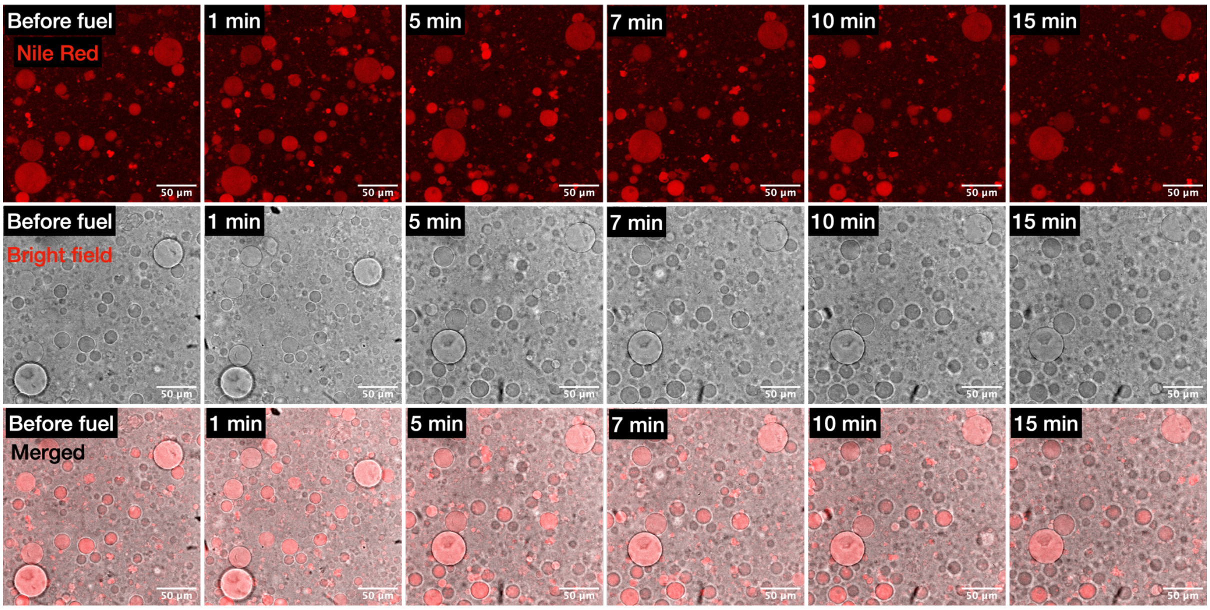

Figure S13. Fluorescence and bright-field microscopy analysis of mixed C10 vesicles post-EDC addition. C10 vesicles (65 mM) at pH 4.94 in 200 mM MES buffer were fueled 10 mM EDC. Two distinct vesicle populations were prepared: one labelled with 2 μM Nile Red and another unlabeled. The two populations were carefully mixed and imaged using fluorescence and bright-field microscopy to assess potential vesicle fusion induced by EDC. The top panel presents fluorescence images of Nile Red-stained vesicles at different time points post-EDC addition. The middle panel shows corresponding bright-field microscopy images of the vesicle populations. In contrast, the bottom panel provides the merged fluorescence and bright-field microscopy images, highlighting the spatial relationship between the labelled and unlabeled vesicles. No vesicle fusion was observed at this scale over the time points examined.

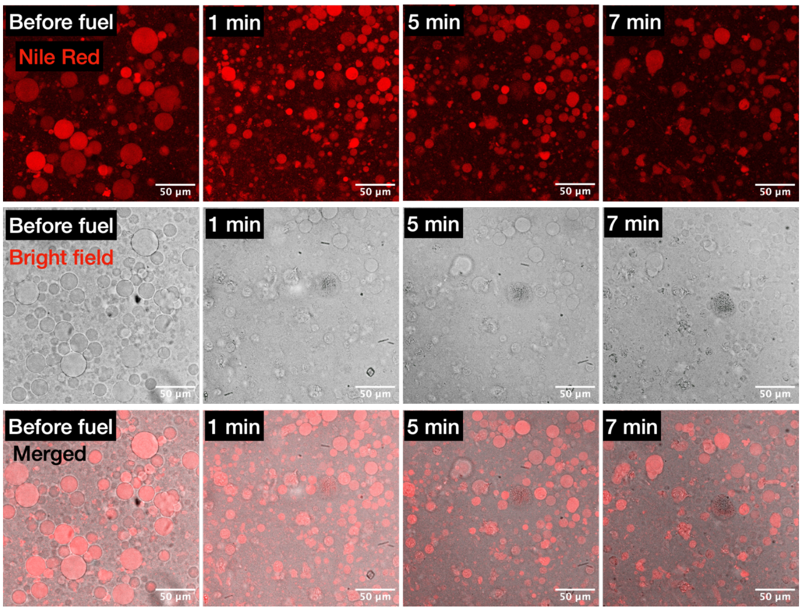

Fig. S14. C10 vesicles (65 mM) at pH 4.94 in 200 mM MES buffer were subjected to the addition of 23 mM EDC, followed by manual agitation (up-and-down pipetting) to homogenize the sample. Two distinct vesicle populations were prepared: one labeled with 2 µM Nile Red and another unlabeled. The mixed populations were imaged using fluorescence and bright-field microscopy to assess potential vesicle fusion induced by EDC under these conditions. The top panel presents fluorescence images of Nile Red-stained vesicles at various time points post-EDC addition, while the middle panel displays the corresponding bright-field microscopy images. The bottom panel shows merged images of fluorescence and bright-field microscopy.

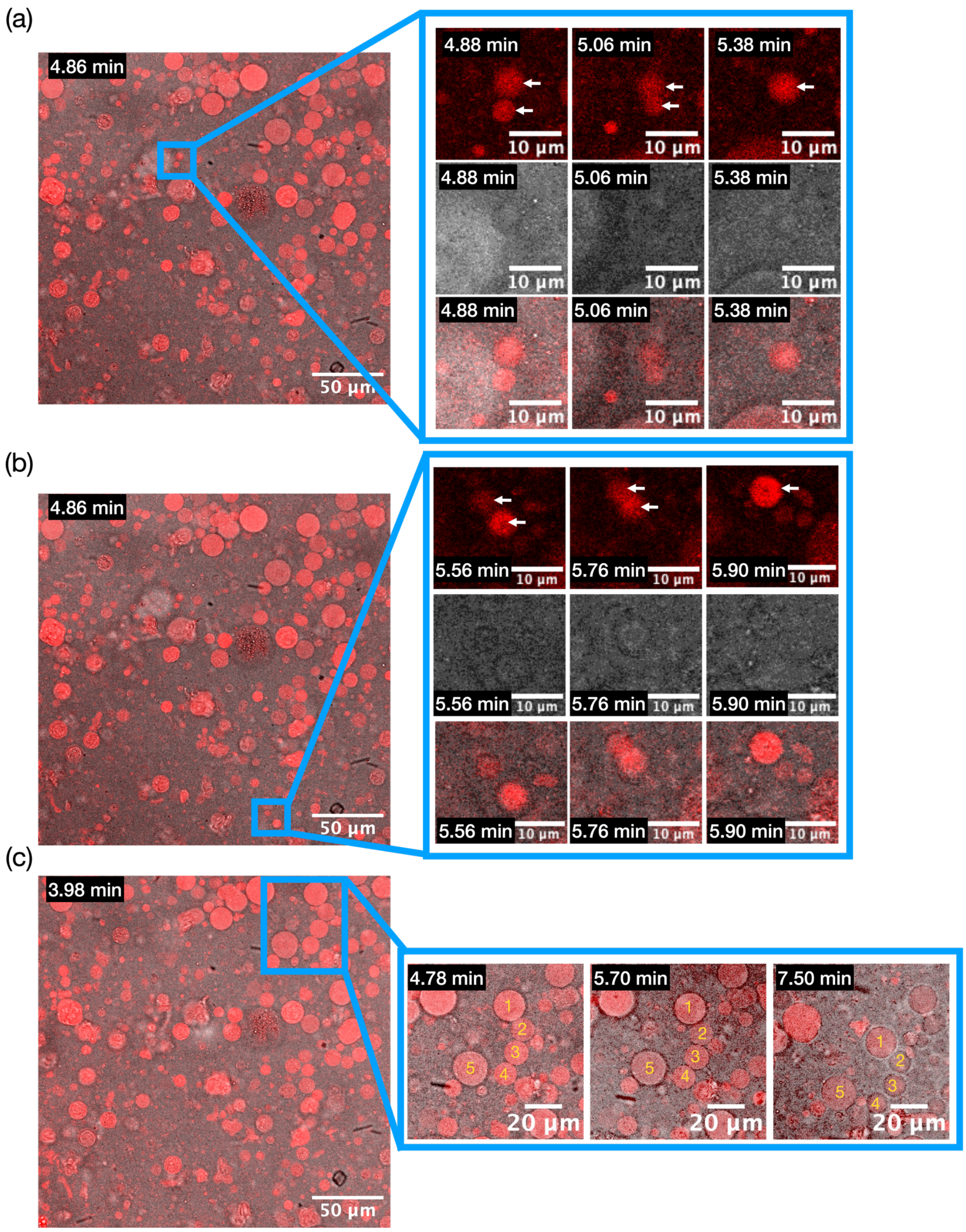

Figure S15: Close-up fluorescence and bright-field microscopy analysis of C10 vesicles after adding 23 mM EDC with agitation. C10 vesicles (65 mM) at pH 4.94 in 200 mM MES buffer, and Nile Red 2 µM, were subjected to the addition of 23 mM EDC, followed by manual agitation (up-and-down pipetting) to homogenize the sample. (a) and (b) show the details of small vesicles fusion after adding EDC. On the other hand, (c) shows that larger-diameter vesicles do not fuse.

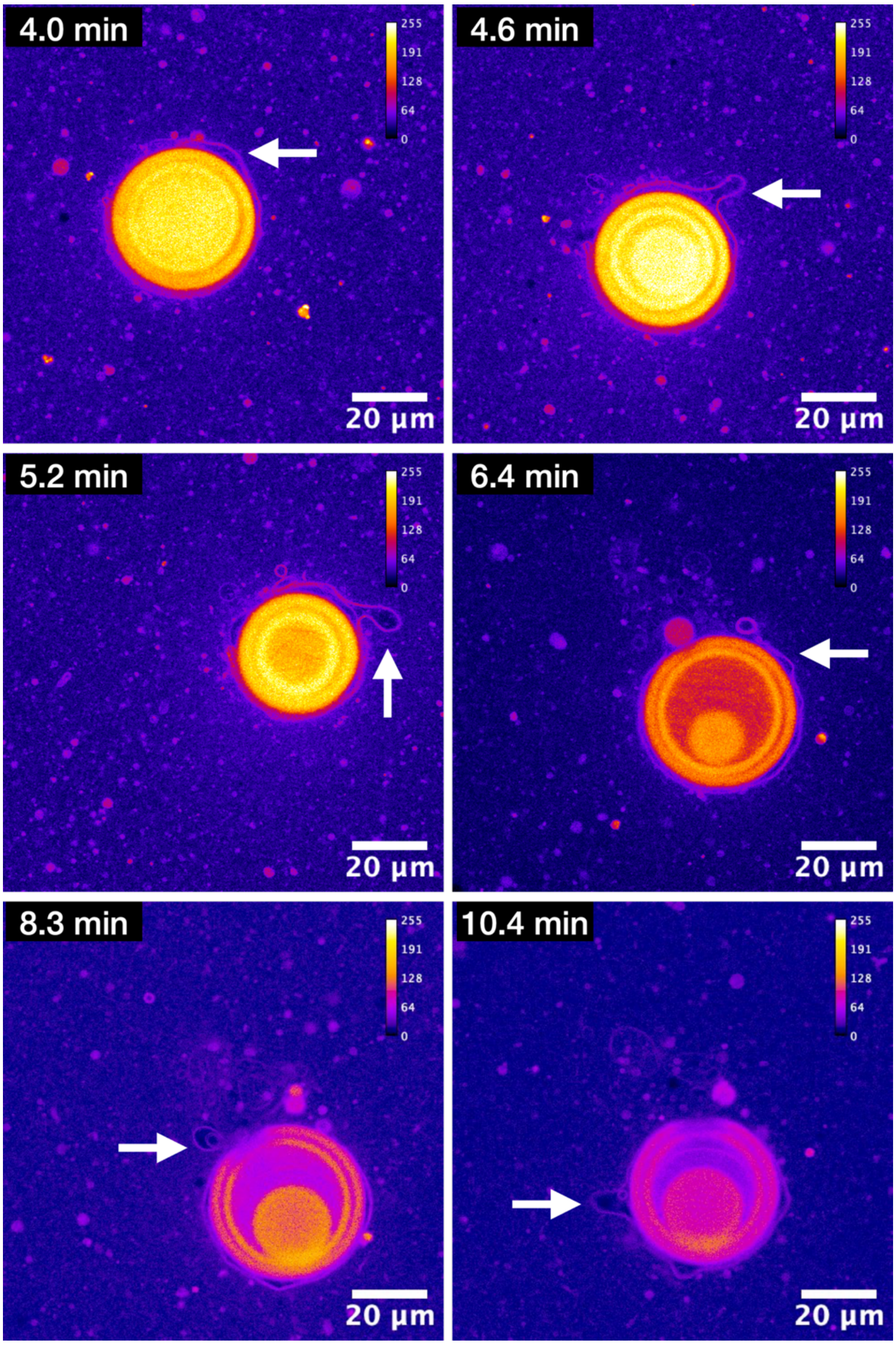

Figure S16. Time-series confocal micrographs of a C10 multilamellar vesicle (65 mM, 200 mM MES, pH 4.9) after addition of EDC 10 mM. White arrow indicates the emergence of new membranous structures. Dye: Nile red 2 µM. Images obtained from Movie S6.

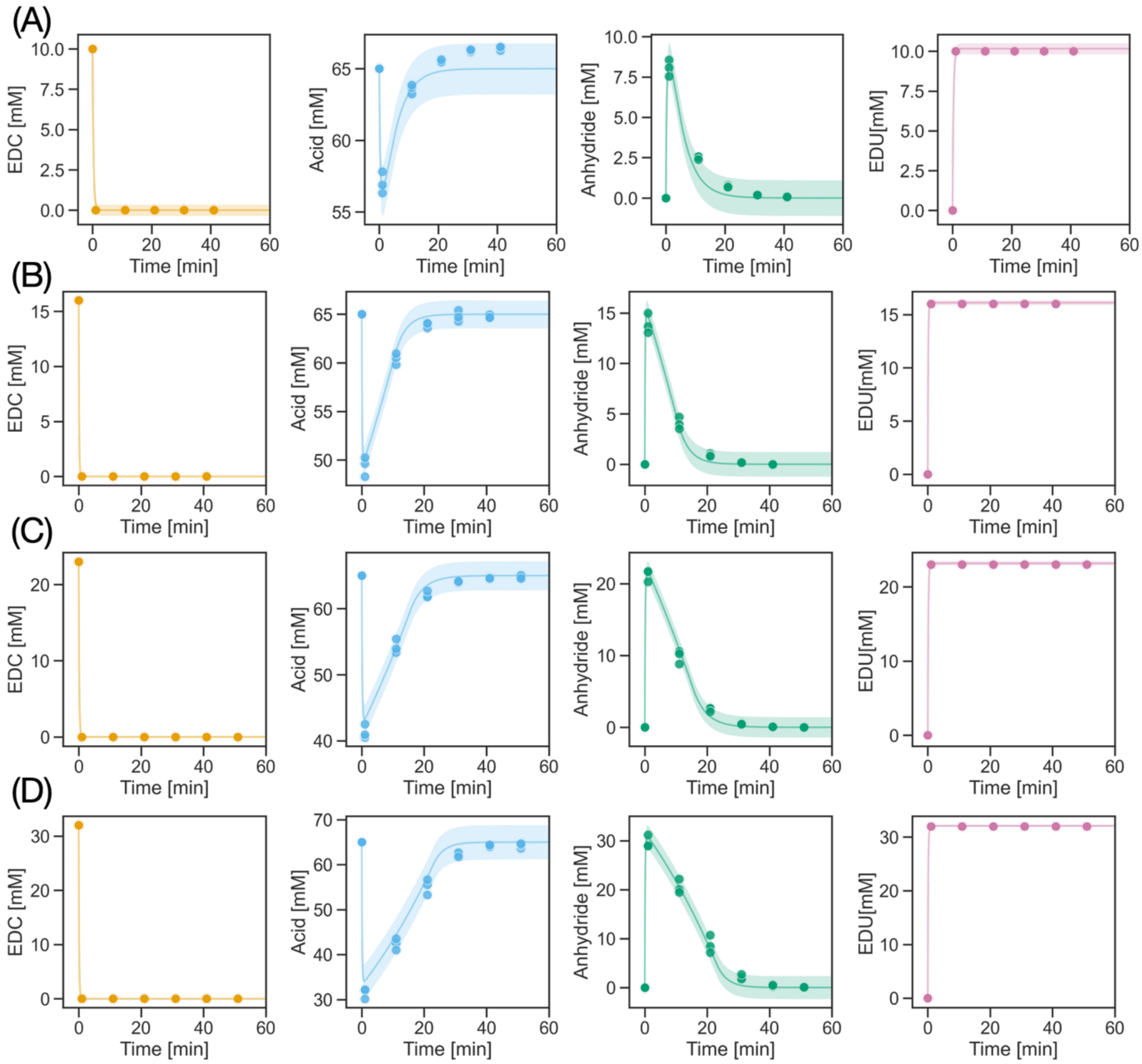

Figure S17. HPLC data (dots) and kinetic model (line) of C10 65 mM in 200 mM MES buffer and pH 4.94 in the presence of (a) 10 mM EDC, (b) 16 mM EDC, (c) 23 mM EDC and (d) 32 mM EDC. Data for precursor, anhydride, EDC and waste (W, EDU) are plotted. All HPLC experiments were performed in triplicate.

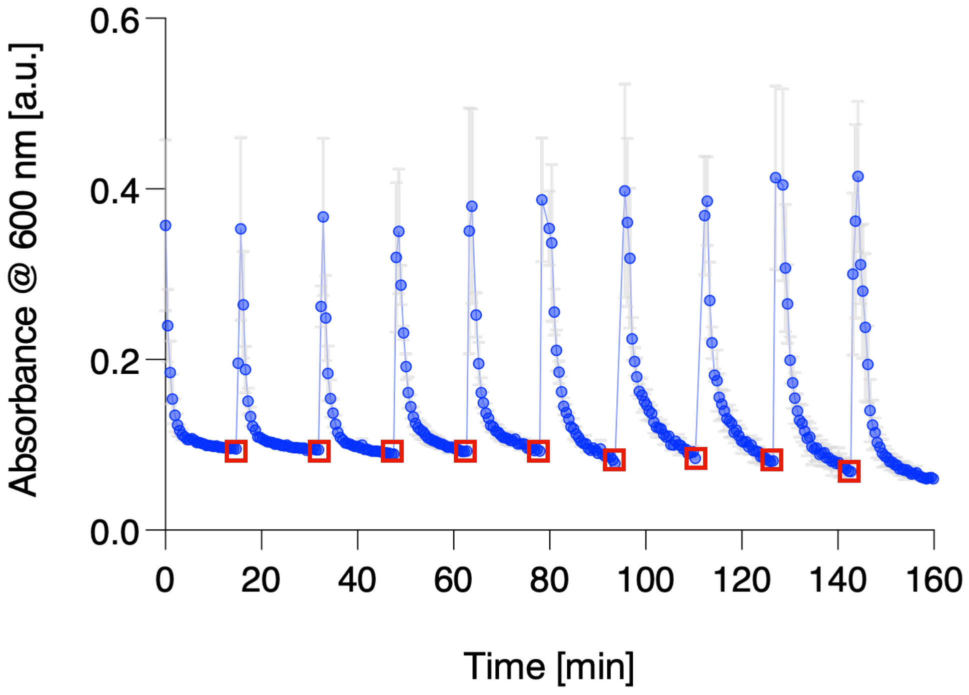

Figure S18. Iterative fueling of 65 mM C10 multilamellar vesicles in 200 mM MES buffer at pH 4.94 with 10 mM EDC. Red squares indicate the time points of EDC injections, administered at 15-minute intervals. Error bars represent the standard deviation from three independent experiments.

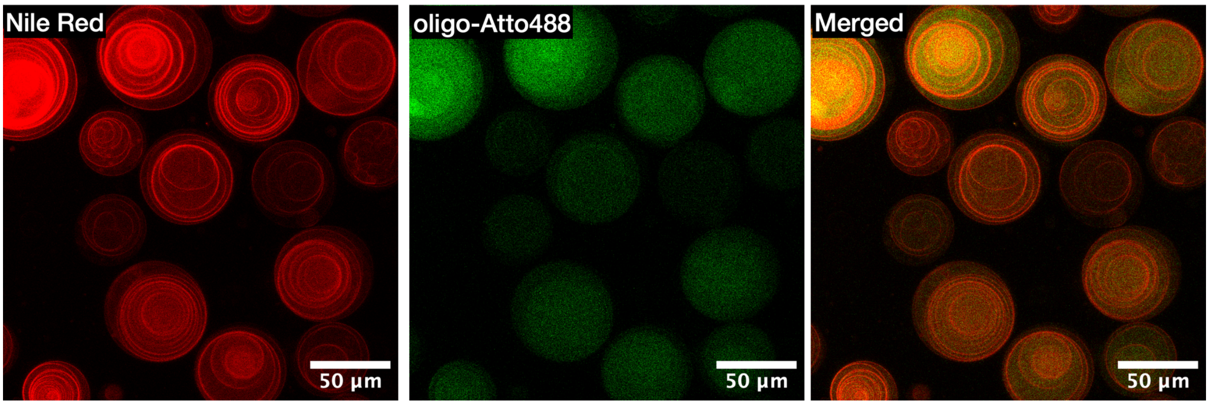

Figure S19. Confocal micrographs of C10 multilamellar vesicles (65 mM, MES buffer 200 mM, pH 4.9) with encapsulated labeled-DNA. Dyes: Nile Red 2 μM (bilayer; red color) and labeled DNA ATTO488 1.5 μM (green color).

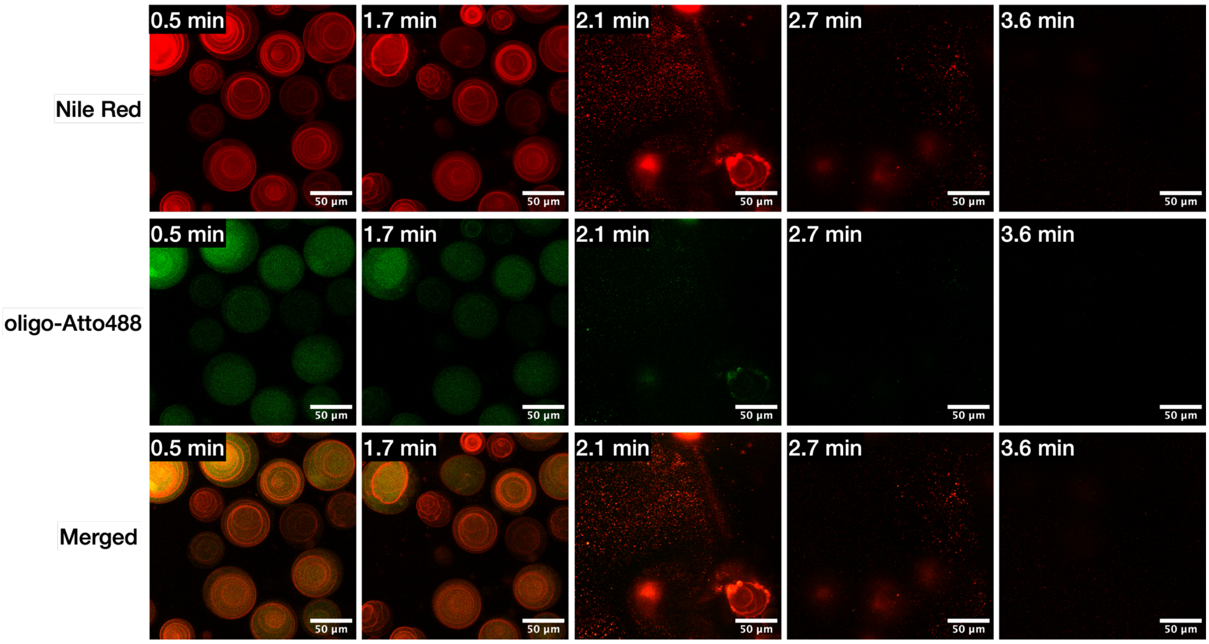

Figure S20. Collapse of C10 multilamellar vesicles upon exposure to 65 mM EDC and release of encapsulated oligo-Atto488. The images show the rapid collapse of 65 mM C10 multilamellar vesicles at pH 4.94 in 200 mM MES buffer, encapsulating 4 μM oligo-Atto488, following the overexposure to 65 mM EDC. The process was monitored during the vesicle collapse's initial minutes as they converted into anhydride oil droplets. The top row shows the fluorescence of Nile Red-stained (2 μM) vesicle membranes at various time points. The middle row displays the encapsulated oligo-Atto488, progressively released into the solution as the vesicles collapse without binding to the membrane. The bottom row presents merged images of both fluorescence channels, demonstrating the spatial relationship between the membrane collapse and oligo-DNA release into the surrounding solution.

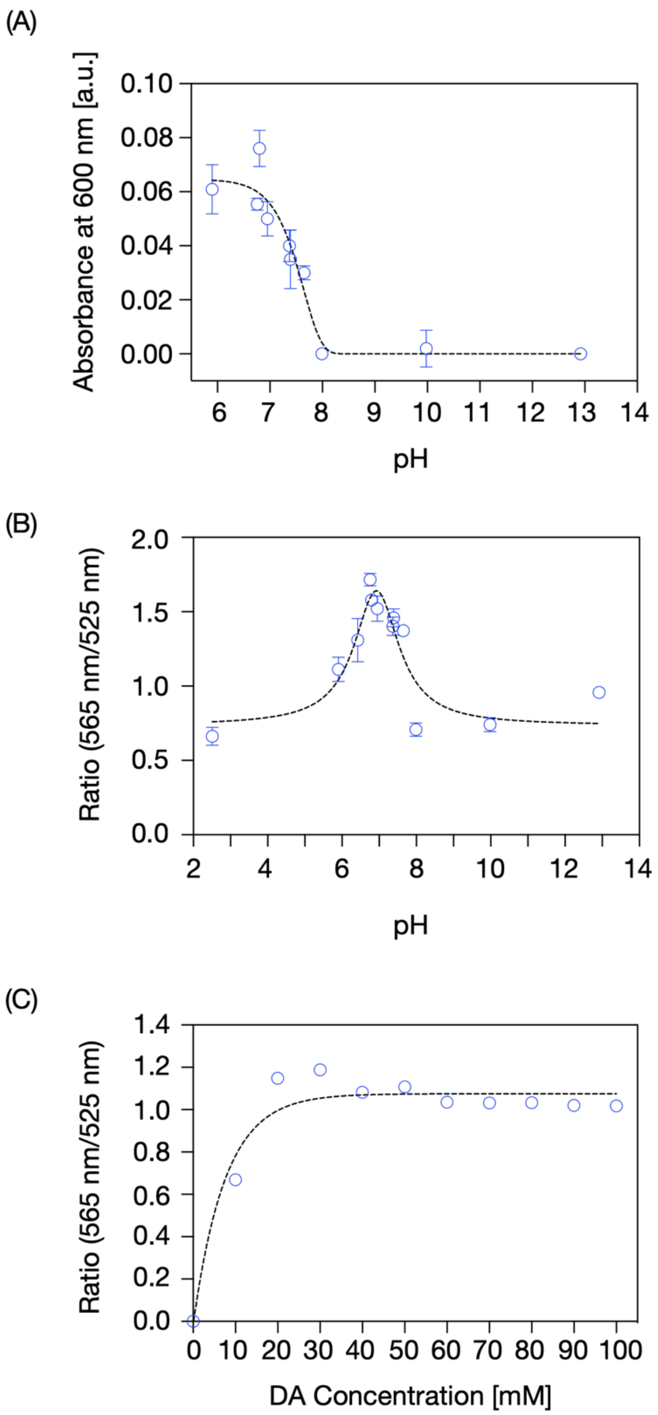

Figure S21. (A) Plot of absorbance at 600 nm of DA 50 mM in MES buffer vs pH. Vesicles are present at values close to the inflexion point of the curve. **(B)** Absorbance ratio (A565 nm/A525 nm) changes in response to pH variations, determined using merocyanine 540 as a lipophilic probe sensitive to lipid packing. (**C**) Variation in the absorbance ratio (A565 nm/A525 nm) with different DA concentrations, indicating the critical vesicle concentration (CVC ~40 mM) using merocyanine 540. All merocyanine 540-containing samples were equilibrated in the dark before measurement. Error bars are standard deviations (SD) with n = 3.

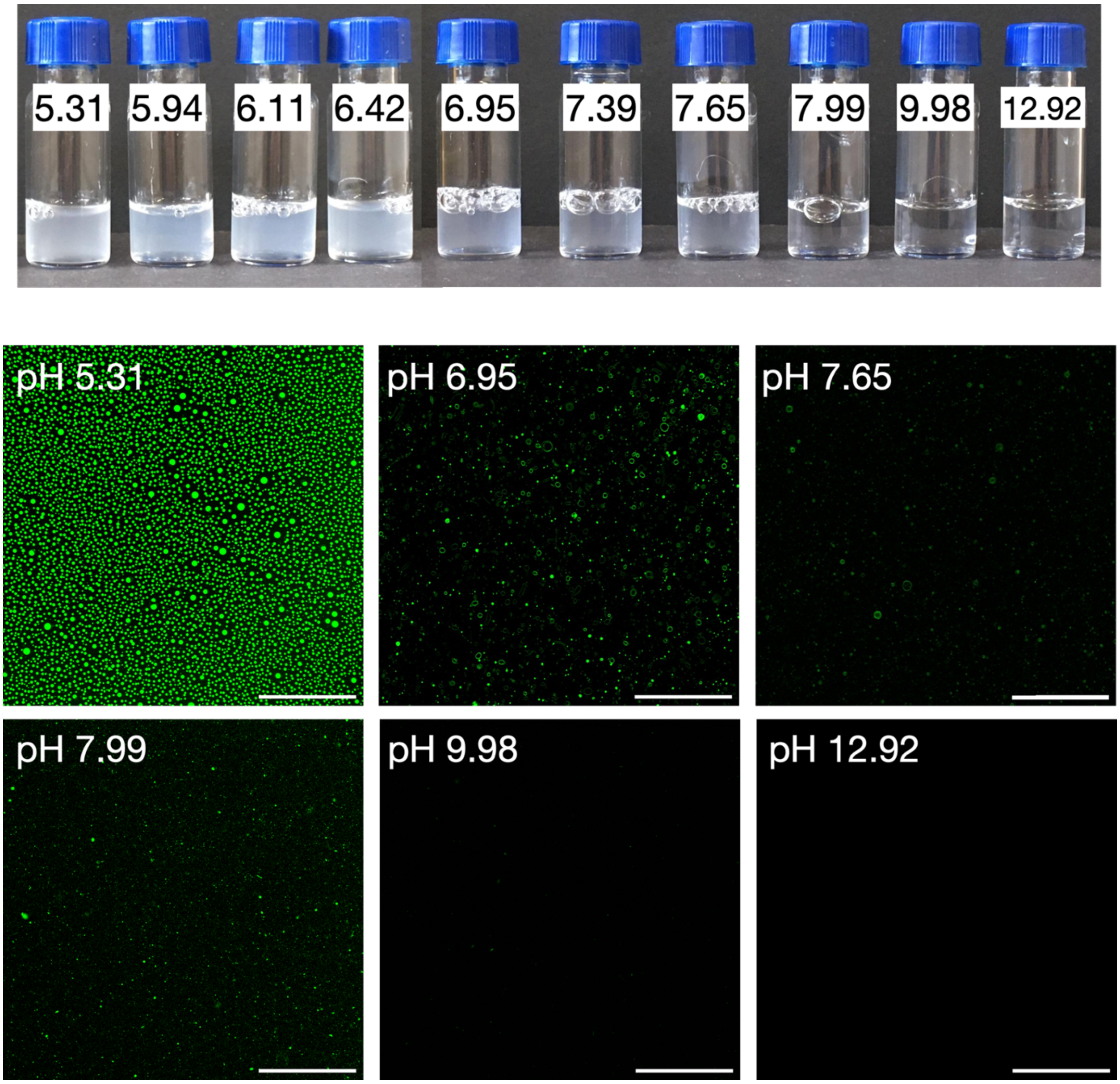

Figure S22. (Top panel) Visual appearance of DA solutions (50 mM) vs pH. (Bottom panel) Confocal micrographs of DA (50 mM) at different pH values (after 24 h). Dye: Nile Red 2 µM. Scale bar: 20 µm.

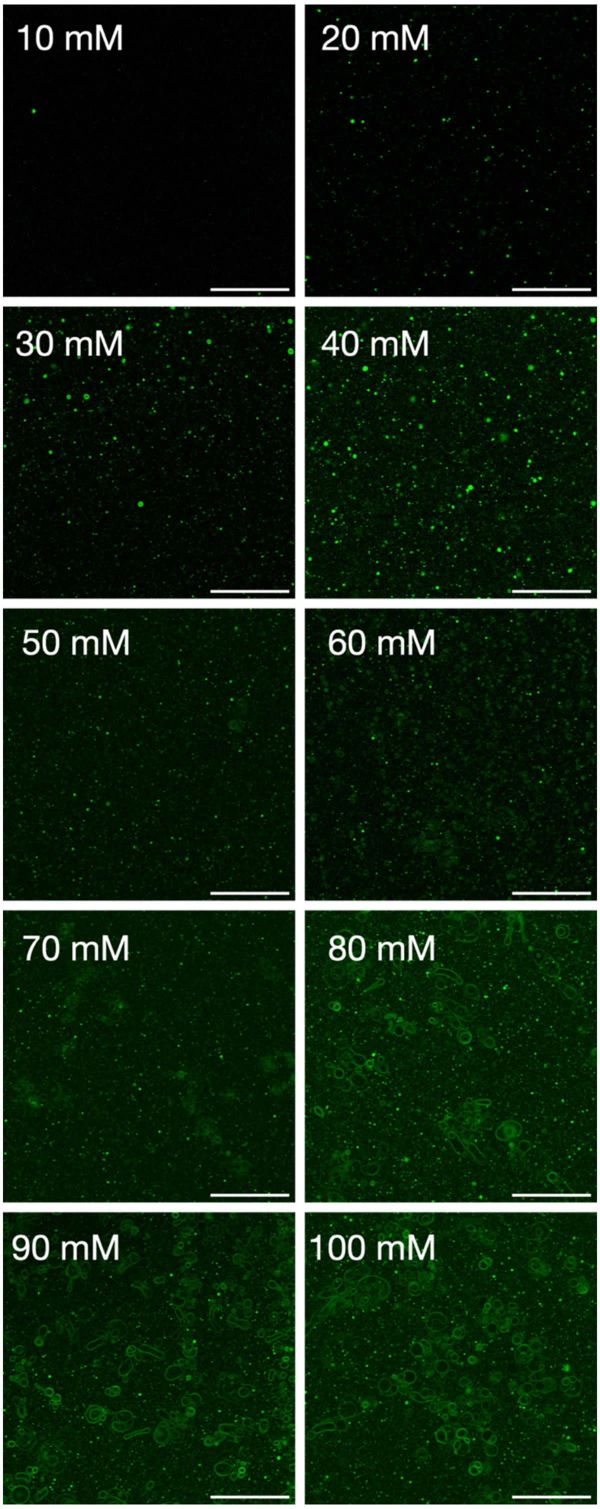

Figure S23. Confocal micrographs of DA at different concentrations (pH 6.81) after 30 min of hydration. Dye: Nile Red 2 μM. Scale bar: 20 μm.

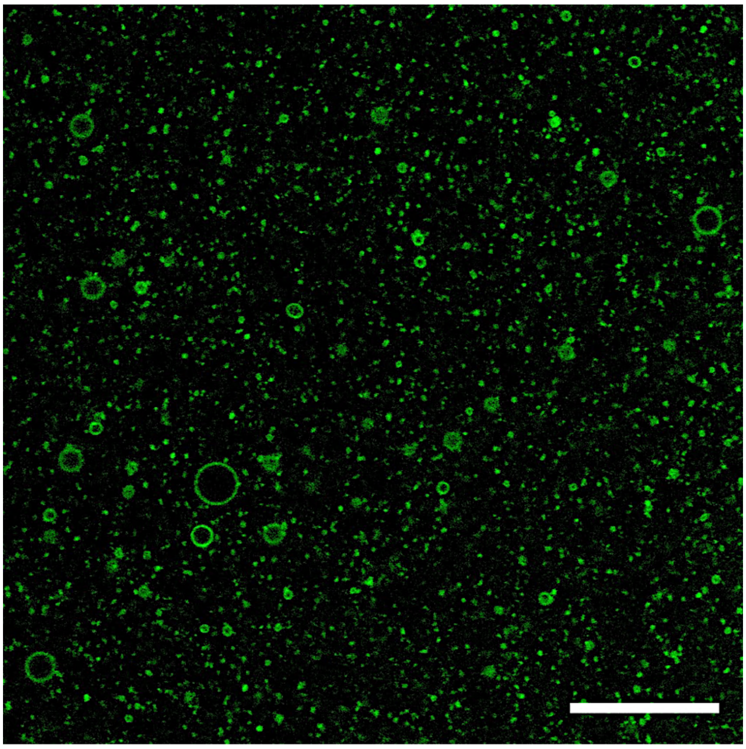

Figure S24. Representative confocal micrograph of DA (50 mM in MES buffer 200 mM, pH 6.8). Dye: Nile Red 2 μM. Scale bar: 50 μm.

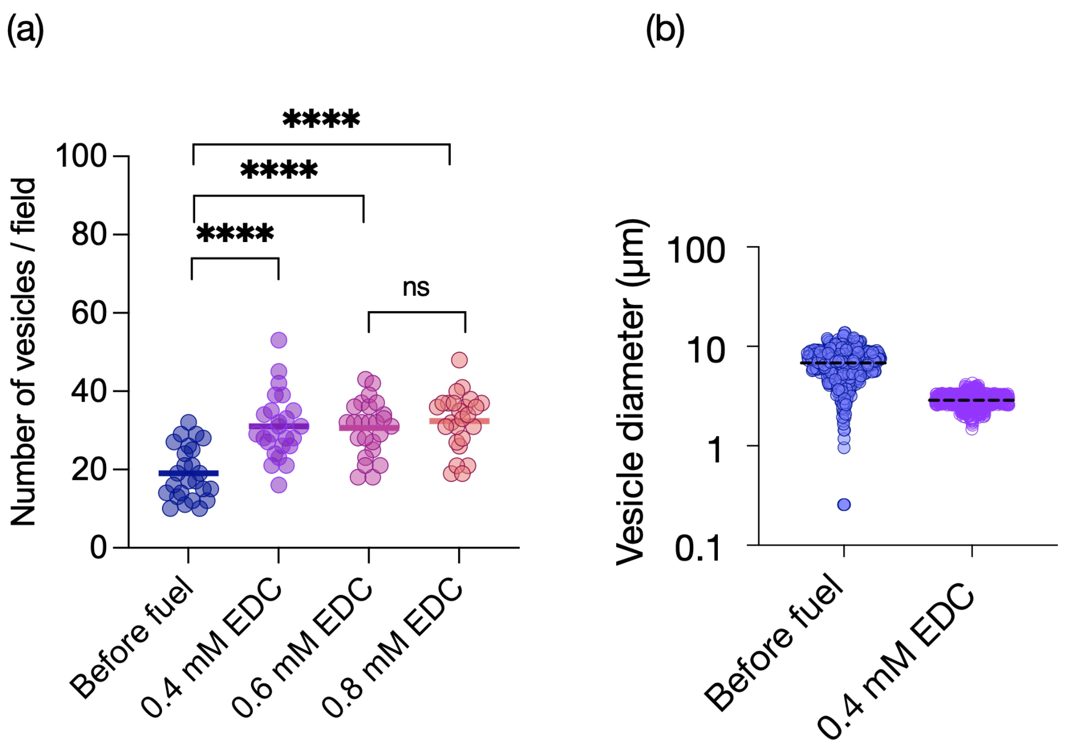

Figure S25. (a) Quantification of vesicle counts per field across varying EDC concentrations. Decanoic acid vesicle counts per field were distributed under four experimental conditions (n = 25 for each condition). The mean vesicle count in the "Before fuel" condition was 19.12 ± 6.33, which increased significantly in the 0.4 mM EDC group (mean = 30.36 ± 7.97, ****P < 0.0001), 0.6 mM EDC group (mean = 31.56 ± 6.39, ****P < 0.0001), and 0.8 mM EDC group (mean = 32.44 ± 6.72, ****P < 0.0001). No significant difference (ns) was observed between the 0.6 mM and 0.8 mM EDC groups. Statistical analysis was conducted using one-way ANOVA ($F(3, 96) = 18.14$, $P < 0.0001$), with Brown-Forsythe and Bartlett's tests confirming the homogeneity of variances across groups ($P > 0.05$). These results indicate an increase in vesicle count upon EDC addition, which plateaus at 0.6 mM, suggesting a saturation point beyond which no further increase in vesicle count is observed. (b) Vesicle diameters (µm) before the addition of EDC ("Before fuel," n = 476) and after the addition of 0.4 mM EDC (n = 775), displayed on a logarithmic scale. The median diameter was 7.91 µm for the "Before fuel" group and 4.63 µm for the 0.4 mM EDC group, with a narrower distribution observed after EDC addition. Statistical tests confirmed non-normality in both groups: D'Agostino & Pearson (K2 = 426.8 and 31.68; P < 0.0001 for both, ***), Anderson-Darling (A2 = 6.825 and 2.831; P < 0.0001, ****), Shapiro-Wilk (W = 0.9449 and 0.9800; P < 0.0001, ****), and Kolmogorov-Smirnov (KS = 0.07868 and 0.04308; P < 0.0001 and P = 0.0017, **** and **). These findings indicate significant deviations from normality, justifying non-parametric analyses.

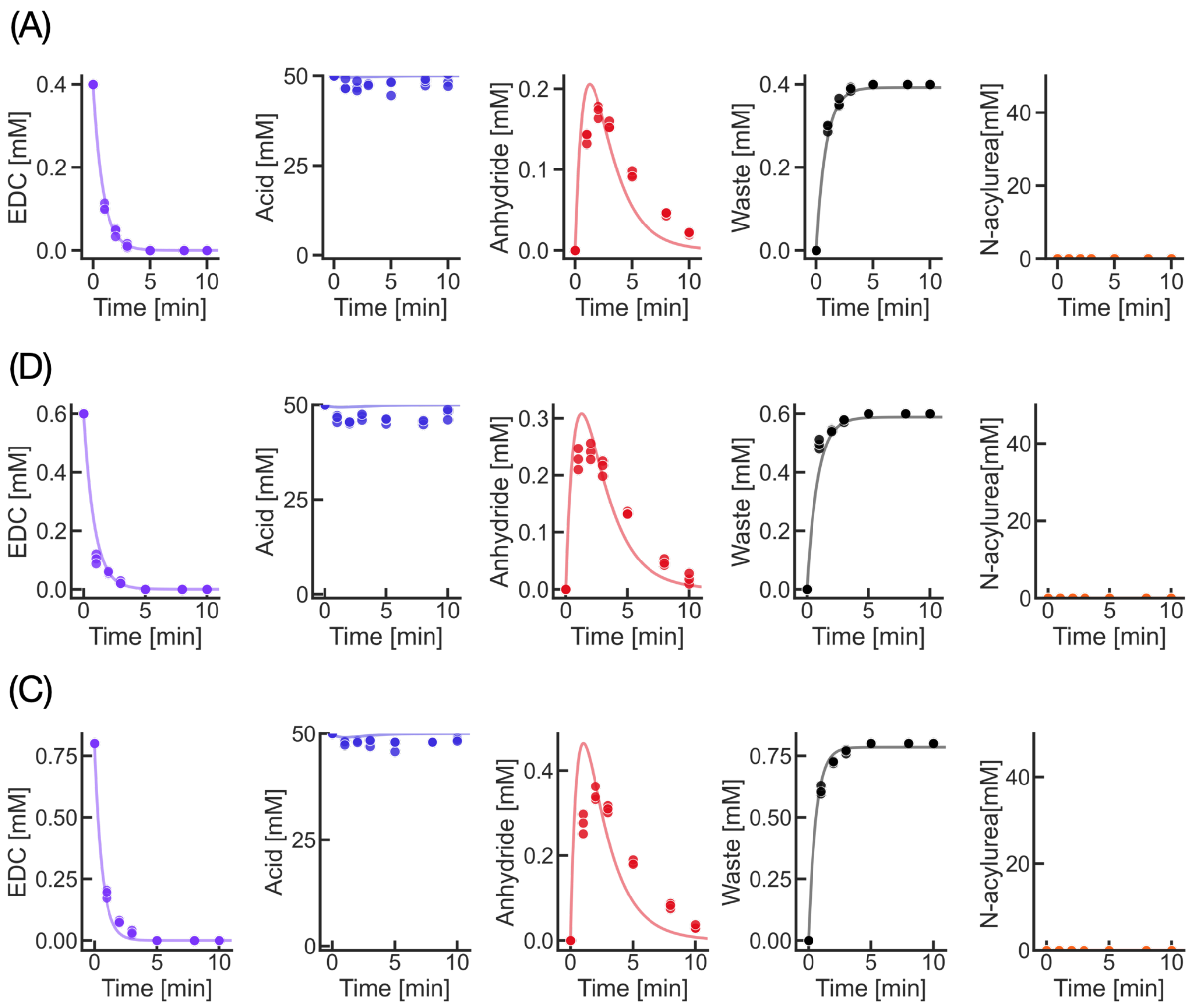

Figure S26. HPLC data (dots) and kinetic model (line) for decanoic acid vesicles (50 mM) at pH 6.8 with EDC concentrations of (A) 0.4 mM, (B) 0.6 mM, and (C) 0.8 mM. Data for precursor, anhydride, EDC, and waste (W, EDU) are plotted. All HPLC experiments were performed in triplicate.

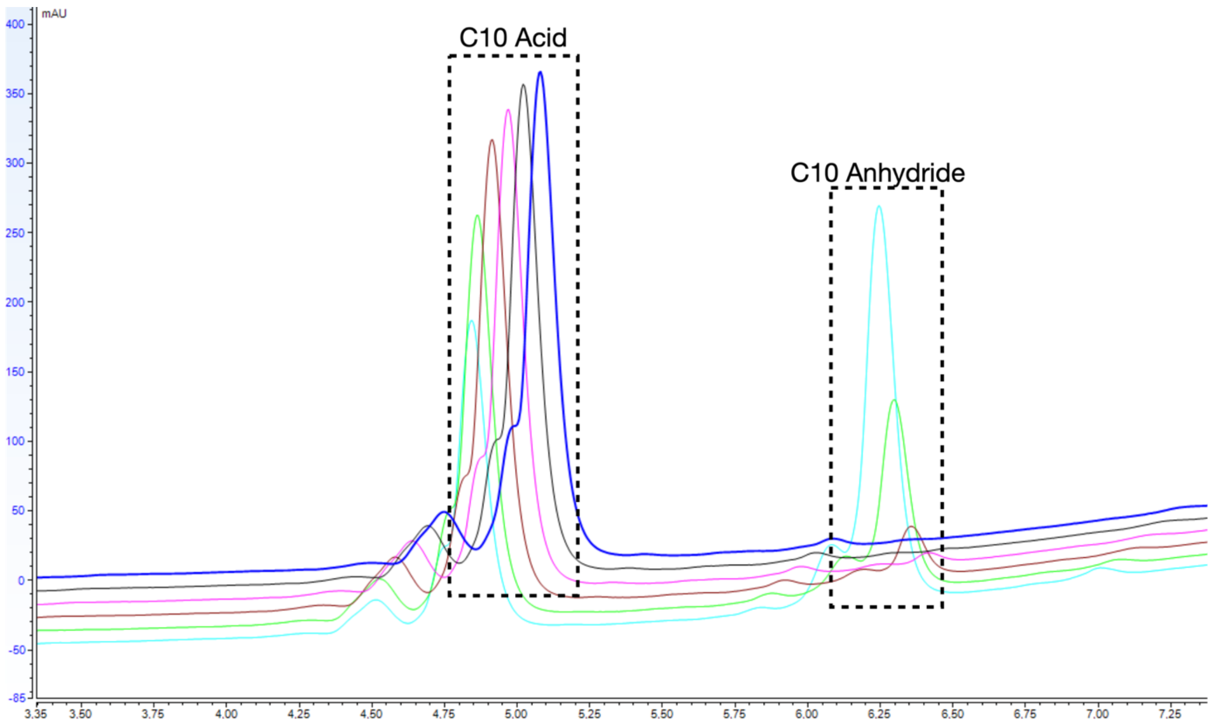

Figure S27. HPLC chromatograms illustrating the kinetics of C10 65 mM (acidic form) in MES 200 mM, pH 4.9 following the addition of 23 mM EDC. Immediately after the addition of EDC, a prominent peak corresponding to the anhydride is observed (cyan peak). The chromatograms display the temporal progression of anhydride formation on the right and acid formation on the left. The series represent different time points: 1 min (cyan), 11 min (green), 21 min (brown), 31 min (purple), 41 min (black), and 51 min (blue).

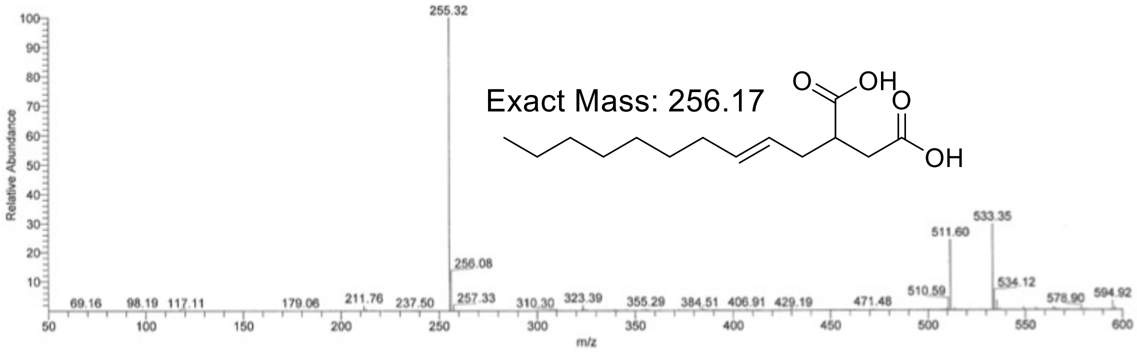

Figure S28. Mass spectrum of the precursor (C10 acid).

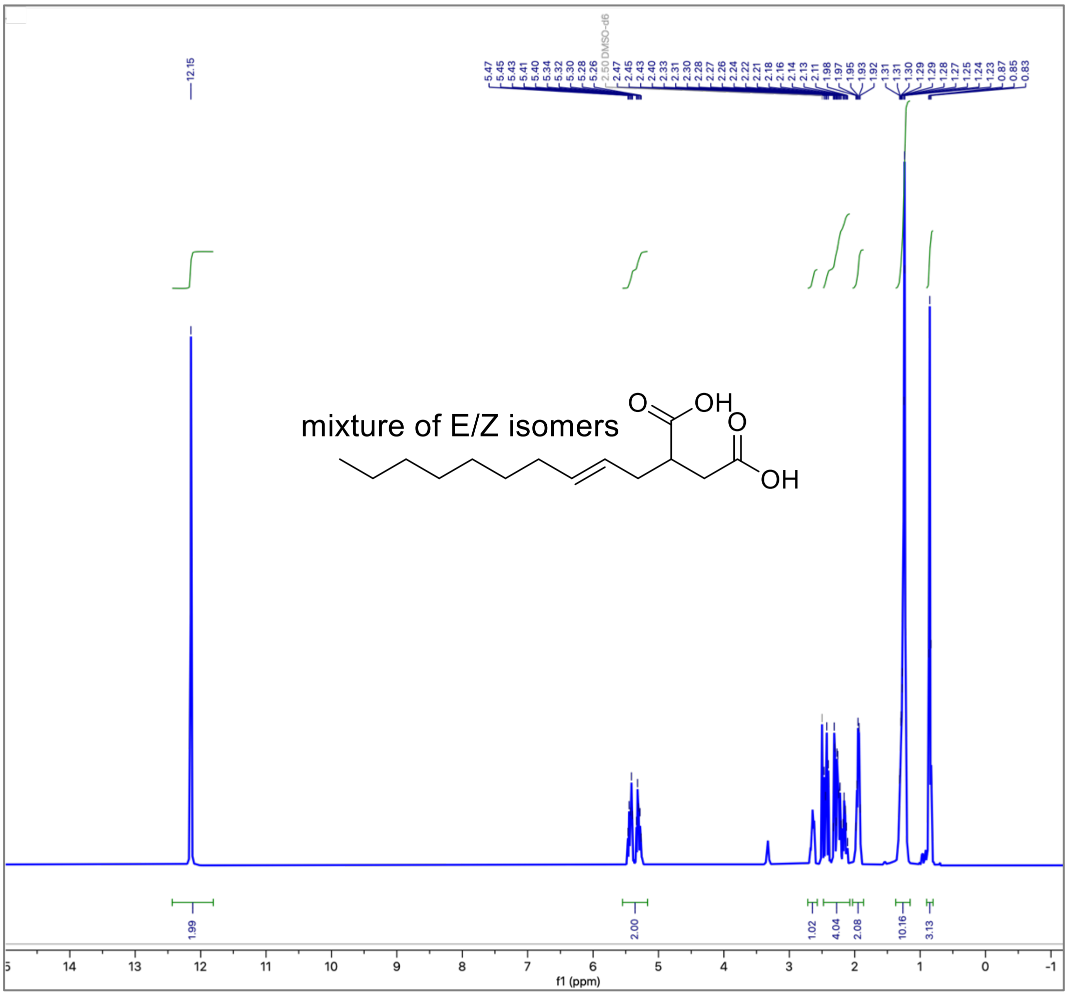

Figure S29. ^{1}H NMR (400 MHz, DMSO) spectrum of the precursor (C10 acid).

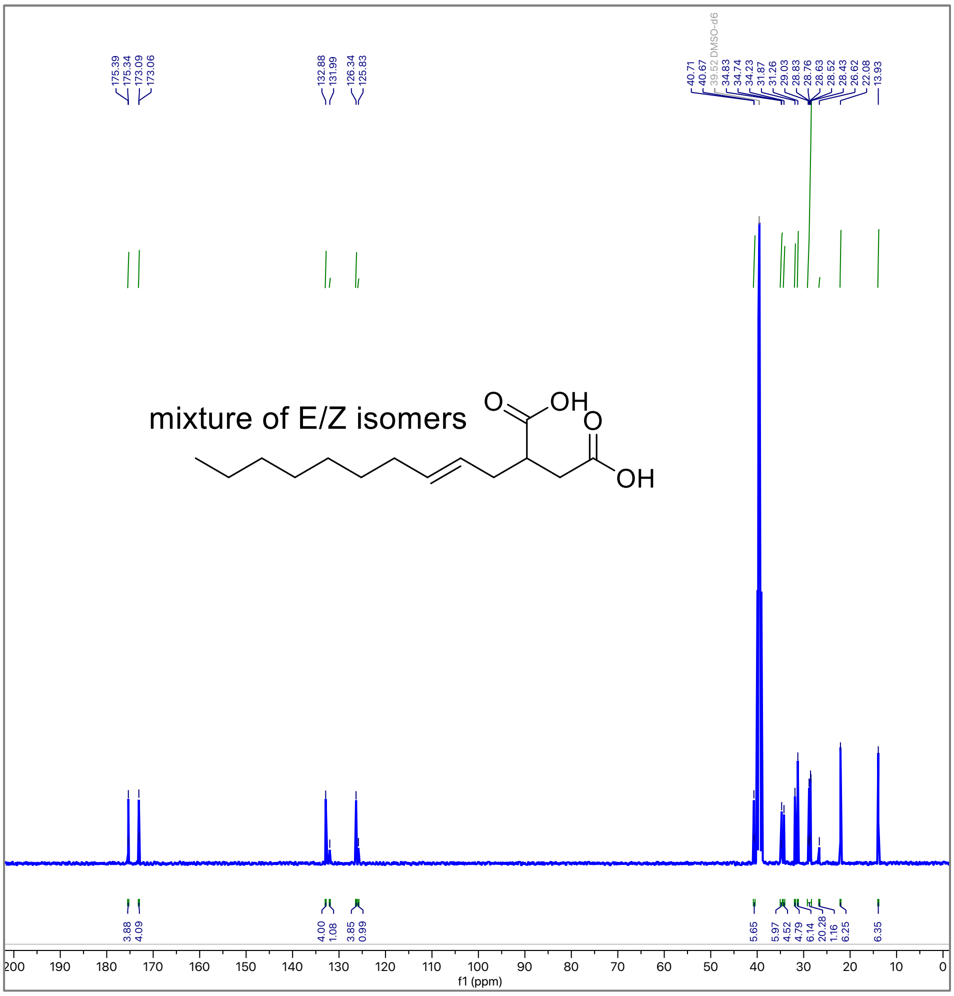

Figure S30. ^{13}C NMR (101 MHz, DMSO) spectrum of the precursor (C10 acid).

Description of Supplementary Movies

Movie S1. Morphological transition of a C10 multilamellar vesicle (65 mM) in response to 23 mM EDC at pH 4.9. Vesicles were stained with 2 μM Nile Red to highlight structural features. Scale bar: 20 μm.

Movie S2. Time-dependent morphological transition of 65 mM C10 multilamellar vesicles in 200 mM MES buffer at pH 4.94 following the addition of 23 mM EDC. Vesicles were stained with 2 μM Nile Red.

Movie S3. Formation and division process of "daughter" vesicles from the surface of a multilamellar vesicle. Conditions: C10 65 mM precursor with 2 μM Nile Red dye, 10 mM EDC, and 200 mM MES buffer at pH 4.94.

Movie S4. Formation of an elongated “daughter" vesicle from the surface of a multilamellar vesicle. Conditions: C10 65 mM precursor with 2 μM Nile Red dye, 10 mM EDC, and 200 mM MES buffer at pH 4.94.

Movie S5. Membrane formation on the surface of a C10 multilamellar vesicle (65 mM) in 200 mM MES buffer at pH 4.9 after adding 10 mM EDC. Vesicles were stained with 2 μM Nile Red.

Movie S6. Division process of a multilamellar vesicle labeled with 2 μM Nile Red and containing encapsulated material (1.5 μM labeled DNA) in the presence of 10 mM EDC. The sequence reveals the formation of smaller daughter vesicles while maintaining the integrity of the encapsulated material. Scale bar: 10 μm.

Movie S7. Division sequence of decanoic acid (DA) vesicles (50 mM) at pH 6.8 upon exposure to 0.4 mM EDC. Over time, the vesicle structure becomes disrupted, leading to the formation of smaller daughter vesicles. Vesicles were stained with 2 μM Nile Red. Scale bar: 10 μm.

References.

(1) Moga, A.; Yandrapalli, N.; Dimova, R.; Robinson, T. Optimization of the Inverted Emulsion Method for High-Yield Production of Biomimetic Giant Unilamellar Vesicles. *ChemBioChem* **2019**, *20* (20). https://doi.org/10.1002/cbic.201900529.

(2) Monnard, P. A.; Deamer, D. W. Preparation of Vesicles from Nonphospholipid Amphiphiles. *Methods Enzymol* **2003**, *372*. https://doi.org/10.1016/S0076-6879(03)72008-4.

(3) Bonfio, C.; Russell, D. A.; Green, N. J.; Mariani, A.; Sutherland, J. D. Activation Chemistry Drives the Emergence of Functionalised Protocells. *Chem Sci* **2020**, *11* (39). https://doi.org/10.1039/d0sc04506c.

(4) Wanzke, C.; Tena-Solsona, M.; Rieß, B.; Tebcharani, L.; Boekhoven, J. Active Droplets in a Hydrogel Release Drugs with a Constant and Tunable Rate. *Mater Horiz* **2020**, *7* (5). https://doi.org/10.1039/c9mh01822k.

(5) Namani, T.; Walde, P. From Decanoate Micelles to Decanoic Acid/Dodecylbenzenesulfonate Vesicles. *Langmuir* **2005**, *21* (14). https://doi.org/10.1021/la047028z.

(6) Williamson, P.; Mattocks, K.; Schlegel, R. A. Merocyanine 540, a Fluorescent Probe Sensitive to Lipid Packing. *BBA - Biomembranes* **1983**, *732* (2). https://doi.org/10.1016/0005-2736(83)90055-X.

(7) Gans, P.; Sabatini, A.; Vacca, A. Investigation of Equilibria in Solution. Determination of Equilibrium Constants with the HYPERQUAD Suite of Programs. *Talanta* **1996**, *43* (10). https://doi.org/10.1016/0039-9140(96)01958-3.

(8) Göpfrich, K.; Haller, B.; Staufer, O.; Dreher, Y.; Mersdorf, U.; Platzman, I.; Spatz, J. P. One-Pot Assembly of Complex Giant Unilamellar Vesicle-Based Synthetic Cells. *ACS Synth Biol* **2019**, *8* (5). https://doi.org/10.1021/acssynbio.9b00034.

(9) Schindelin, J.; Arganda-Carreras, I.; Frise, E.; Kaynig, V.; Longair, M.; Pietzsch, T.; Preibisch, S.; Rueden, C.; Saalfeld, S.; Schmid, B.; Tinevez, J. Y.; White, D. J.; Hartenstein, V.; Eliceiri, K.; Tomancak, P.; Cardona, A. Fiji: An Open-Source Platform for Biological-Image Analysis. *Nature Methods*. 2012. https://doi.org/10.1038/nmeth.2019.

(10) Richter, K. N.; Revelo, N. H.; Seitz, K. J.; Helm, M. S.; Sarkar, D.; Saleeb, R. S.; D'Este, E.; Eberle, J.; Wagner, E.; Vogl, C.; Lazaro, D. F.; Richter, F.; Coy-Vergara, J.;

Coceano, G.; Boyden, E. S.; Duncan, R. R.; Hell, S. W.; Lauterbach, M. A.; Lehnart, S. E.; Moser, T.; Outeiro, T. F.; Rehling, P.; Schwappach, B.; Testa, I.; Zapiec, B.; Rizzoli, S. O. Glyoxal as an Alternative Fixative to Formaldehyde in Immunostaining and Super-resolution Microscopy. *EMBO J* **2018**, *37* (1). https://doi.org/10.15252/embj.201695709.

(11) Sheppard, C. J. R. The Development of Microscopy for Super-Resolution: Confocal Microscopy, and Image Scanning Microscopy. *Applied Sciences (Switzerland)*. 2021. https://doi.org/10.3390/app11198981.

(12) Chuo, S. T. Y.; Chien, J. C. Y.; Lai, C. P. K. Imaging Extracellular Vesicles: Current and Emerging Methods. *Journal of Biomedical Science*. 2018. https://doi.org/10.1186/s12929-018-0494-5.

(13) Chen, X.; Soria-Carrera, H.; Zozulia, O.; Boekhoven, J. Suppressing Catalyst Poisoning in the Carbodiimide-Fueled Reaction Cycle. *Chem Sci* **2023**, *14* (44). https://doi.org/10.1039/d3sc04281b.

(14) Kariyawasam, L. S.; Hartley, C. S. Dissipative Assembly of Aqueous Carboxylic Acid Anhydrides Fueled by Carbodiimides. *J Am Chem Soc* **2017**, *139* (34). https://doi.org/10.1021/jacs.7b06099.

(15) Chen, X.; Stasi, M.; Rodon-Fores, J.; Großmann, P. F.; Bergmann, A. M.; Dai, K.; Tena-Solsona, M.; Rieger, B.; Boekhoven, J. A Carbodiimide-Fueled Reaction Cycle That Forms Transient 5(4H)-Oxazolones. *J Am Chem Soc* **2023**, *145* (12). https://doi.org/10.1021/jacs.3c00273.

(16) Chen, X.; Kriebisch, B. A. K.; Bergmann, A. M.; Boekhoven, J. Design Rules for Reciprocal Coupling in Chemically Fueled Assembly. *Chem Sci* **2023**, *14* (37). https://doi.org/10.1039/d3sc02062b.

(17) Schwarz, P. S.; Tebcharani, L.; Heger, J. E.; Müller-Buschbaum, P.; Boekhoven, J. Chemically Fueled Materials with a Self-Immolative Mechanism: Transient Materials with a Fast on/off Response. *Chem Sci* **2021**, *12* (29). https://doi.org/10.1039/d1sc02561a.
